# Dietary tannic acid promotes intestinal clearance of *C. albicans* by cross-linking hyphal chitosan

**DOI:** 10.1371/journal.ppat.1013596

**Published:** 2025-10-15

**Authors:** Jia Feng, Lu Gao, Lijuan Qiu, Wen Chao, Yu Liu, Ruina Wang, Lan Yan, Yuanying Jiang, Quanzhen Lv

**Affiliations:** 1 Department of Pharmacology, Shanghai Tenth People’s Hospital, Tongji University School of Medicine, Shanghai, China; 2 College of Basic Medical Sciences, Naval Medical University, Shanghai, China; 3 Center for Basic Research and Innovation of Medicine and Pharmacy (MOE), School of Pharmacy, Naval Medical University, Shanghai, China; University of Maryland School of Medicine, UNITED STATES OF AMERICA

## Abstract

Intestinal *Candida* overgrowth is the major cause of candidemia in intensive care patients. The lack of phytogenic bioactive components in parenteral nutrition inspired us to focus on the role of polyphenols in preventing intestinal *Candida albicans* overgrowth. Here, we found that tannic acid (TA), which is abundant in tea, coffee, fruit and vegetables, promoted the clearance of *C. albicans* from feces and increased the survival rate of mice by about 60%. Interestingly, an opposite mechanism of TA-induced hyphal aggregation was observed. The main target of TA was identified as chitosan, which constitutes less than 5‰ of the dry weight of the cell wall. The combination of TA and chitosan effectively reduces the invasion and cytotoxicity of *Candida albicans* hyphae on host cells. As a previously neglected component, chitosan is mainly produced by the chitin deacetylase Cda2. Our results elucidate the essential role of Cda2 in modulating chitin and chitosan levels, as well as in maintaining the stress responses and virulence in *C. albicans*, suggesting its potential as a target for new antifungal therapies. The protective role of TA indicates that a plant-based diet is critical for intestinal defense against *C. albicans* pathogenesis, which may develop into new strategies to prevent candidemia.

## Author summary

Candidemia is primarily caused by a significant prior expansion of pathogenic *Candida* in the gastrointestinal tract. The beneficial factors that prevent *C. albicans* pathogenesis in the gut are not well understood. In this study, we revealed that dietary tannic acids play a crucial role in preventing the overgrowth of *C. albicans* by targeting hyphal chitosan, a polysaccharide rather than protein. Using the gastrointestinal infection model, we demonstrated that tannic acid enhances the clearance of *C. albicans* from feces. In vitro studies revealed the inhibitory effect of tannic acid on *C. albicans* hyphae, as evidenced by experiments with yeast-blocked and hyphae-blocked mutants. The binding of tannic acid to chitosan reduced hyphal invasion effectively, but has no significant effect on cell wall proteins abundance. Using the *cda2*Δ/Δ mutant, we showed that the protective effect of tannin acid is dependent on chitosan and confirmed the crucial role of *CDA2* in the pathogenesis, invasion, and response to environmental stresses. Our study provides a preventive strategy for restraining intestinal *C. albicans* overgrowth and highlights the potential benefits of plant-based diets.

## Introduction

*Candida albicans* is the most common fungal pathogen responsible for invasive candidiasis, leading to approximately 1 million deaths worldwide each year [1]. Systemic *C. albicans* infection has been shown to originate from the gastrointestinal tract, as blood isolates of *C. albicans* exhibit genetic similarities with isolates from stool samples [2]. High-resolution microbiota analysis has shown that allogenic hematopoietic cell transplant patients with systemic *Candida* infection suffered a significant expansion of *Candida* species in the intestinal tract [3]. In addition to causing candidemia, colonization or expansion of *C. albicans* in gastrointestinal tract can also exacerbate inflammatory bowel disease, reduce tumor responses to therapies and exacerbate pulmonary neutrophilia during COVID-19 infection [4,5]. Therefore, inhibition of *C. albicans* overgrowth in the gastrointestinal tract represents a compelling strategy to prevent the invasive candidiasis.

The prophylactic use of antifungals such as azoles, echinocandins and amphotericin B is currently recommended for certain surgical patients. However, it is important to carefully weigh the benefits against the potential risks, including adverse effects, development of drug-resistance, and disruption of intestinal fungal homeostasis [4,6]. Targeted eradication of pathogenic *C. albicans* overgrowth in the gut would provide greater therapeutic benefit. Intensive care unit (ICU) patients are at the highest risk of developing invasive candidiasis. Given the total parenteral nutrition approach in ICU patients, we hypothesized that the lack of plant-based foods may lead to dysbiosis of intestinal fungi. In line with this, *Delavy* et al. have just demonstrated that diet is associated with *C. albicans* expansion, as healthy volunteers who ate between meals and on a low-sodium diet had higher levels of *C. albicans* in their gut [7].

The potential bioactive constituents and their role in regulating the homeostasis and pathogenicity of gut fungi remain largely unknown [8]. Numerous plant extracts have antifungal properties, and their impact on gut fungi should not be ignored [9]. Among them, polyphenols as the most abundant plant-derived bioactive constituents in our daily diet, are required for maintaining gut health and microbiome hemostasis [10]. Many polyphenols showed antifungal activities, such as baicalein, naringin, gallic acid, ellagic acid, and corilagin, but their antifungal targets are still unclear [11]. In this study, we found that a dietary polyphenol, tannic acid (TA), can inhibit hyphal proliferation and clear *C. albicans* from the gut. Interestingly, the primary target of tannic acid is chitosan, which is a polysaccharide rather than a protein.

Chitosan is a neglected component of the *C. albicans* cell wall. Chitin content in the yeast *C. albicans* is low, representing only 2–5% of the dry weight of the cell wall. In hyphal cells, it increases significantly to 10–20%. In particular, chitosan is estimated to be less than 5% of the chitin in *C. albicans* [12]. The low amount of chitosan may be an important reason for its neglect in research. Studies in other fungi have shown that deacetylation of chitin to form chitosan can make the polysaccharide more elastic and protect the cell wall from chitinase digestion [13]. In our study, the absence of the chitin deacetylase *CDA2* led to an almost complete reduction of chitosan on the cell wall. The remarkable changes of the *cda2*Δ/Δ mutant in cell wall components, stress responses and virulence highlight the crucial role of chitosan in *C. albicans* pathogenesis. The overlooked chitosan and its unique combination with tannic acid provide a new strategy for the prevention of systemic *C. albicans* infections originating from the gastrointestinal tract, as well as a new understanding of the high susceptibility of ICU patients to candidemia.

## Results

### TA attenuates gastrointestinal *C. albicans* infection

**Fig 1:**
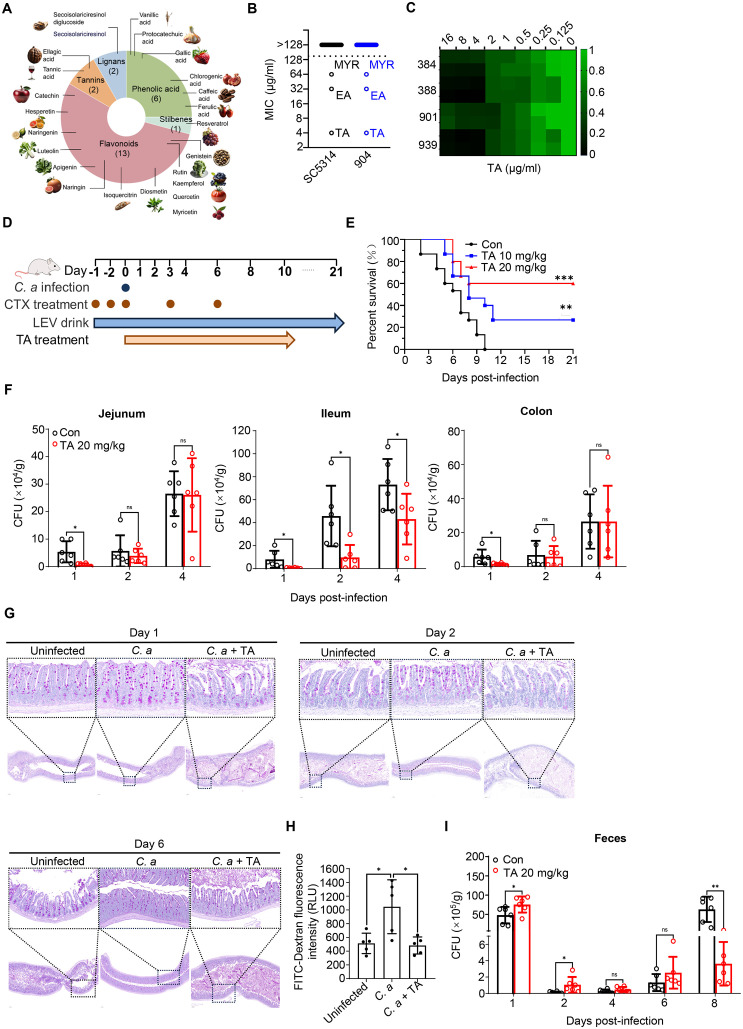
TA protected mice from gastrointestinal *C. albicans* infection. (A) Common dietary polyphenols. (B, C) Minimum inhibitory concentrations (MICs) of 24 dietary polyphenols and Tannic acid (TA) against standard or clinical isolated *C. albicans* 904, 384, 388, 901, 939. (D) Gastrointestinal *C. albicans* infection was induced in female ICR mice by treatment with cyclophosphamide (CTX) and levofloxacin, followed by infection with 4 × 10^8^ CFU/mice of SC5314 and treatment with 10 mg/kg (E) or 20 mg/kg TA (E-H). (E) Survival rates of mice (n = 15). (F) Fungal burden of jejunum, ileum and colon (n = 6). (G) Periodic Acid-Schiff (PAS) staining of ileum (n = 6). Scale bars, top, 20 μm or 50 μm, bottom, 200 μm. (H) Plasma FITC-Dextran fluorescence intensity (n = 5). (I) Fecal fungal burden (n = 6). Data were shown as mean ± SD. Log-rank (Mantel-Cox) test (E), two-tailed unpaired t test (F, H, I). *p < 0.05, **p < 0.01, ***p < 0.001, ns, no significant.

Common polyphenols are classified as flavonoids, phenolic acids, tannins, lignans, and stilbenes (Fig 1A). They are commonly found in vegetables, tea, fruits, and whole grains [14]. To investigate the inhibition of abundant dietary polyphenols on *C. albicans*, the minimum inhibitory concentrations (MICs) of 24 compounds were determined. TA, ellagic acid (EA) and myricetin (MYR) showed activities with MIC ≤ 64 μg/ml against *C. albicans* SC5314 and clinically isolated *C. albicans* 904 (Fig 1B). Among clinically isolated *C. albicans* 384, 388, 901 and 939, TA showed the strongest activity with a MIC of 4 μg/ml (Fig 1C). To investigate the protective effect of TA in the gut, a mouse model of gastrointestinal *C. albicans* infection was employed, using immunosuppressant cyclophosphamide and levofloxacin (Fig 1D). Our results showed that oral administration of TA at 10 mg/kg increased the survival of mice to 30%, while 20 mg/kg of TA increased to 60% (Fig 1E). Consistently, TA treatment reduced the fungal load of jejunum, ileum and colon after infection for 1 day, especially of ileum for 2 and 4 days to lower levels corresponding to those in jejunum or colon (Fig 1F). PAS staining showed that the ileum of mice treated with TA was similar to that of uninfected mice, with a wide lumen and reduced intestinal edema. The muscularis of ileum was thicker in mice infected with *C. albicans* than those treated with TA. Mucin granules in goblet cells, which was stained as purple, were significantly increased in mice infected with *C. albicans*, while decreased by TA treatment. Active goblet cells can produce mucus and uptake intraluminal substances and deliver these antigens to dendritic cells [15]. The increased mucin granules indicated increased intestinal inflammation induced by *C. albicans*. TA treatment inhibited the increase in mucin granules, similar to that observed in uninfected mice, suggesting a less severe intestinal inflammation and infection (Fig 1G). Intestinal permeability was assessed using FITC-Dextran (MW 4000), and the results showed that *C. albicans* infection resulted in significantly higher levels of serum FITC-Dextran, indicating increased intestinal permeability. And serum FITC-Dextran levels in mice treated with TA were similar to those in uninfected mice, suggesting that TA treatment could counteract the damage caused by *C. albicans* (Fig 1H). The fecal fungal load showed that TA treatment increased the early elimination of *C. albicans* from feces and significantly reduced the amount of *C. albicans* in the gut on day 8 post-infection (Fig 1I). These results suggest that TA attenuate the gastrointestinal *C. albicans* infection by promoting the clearance of *C. albicans* from feces.

### TA inhibits the proliferation of hyphae but not yeast-form *C. albicans*

**Fig 2:**
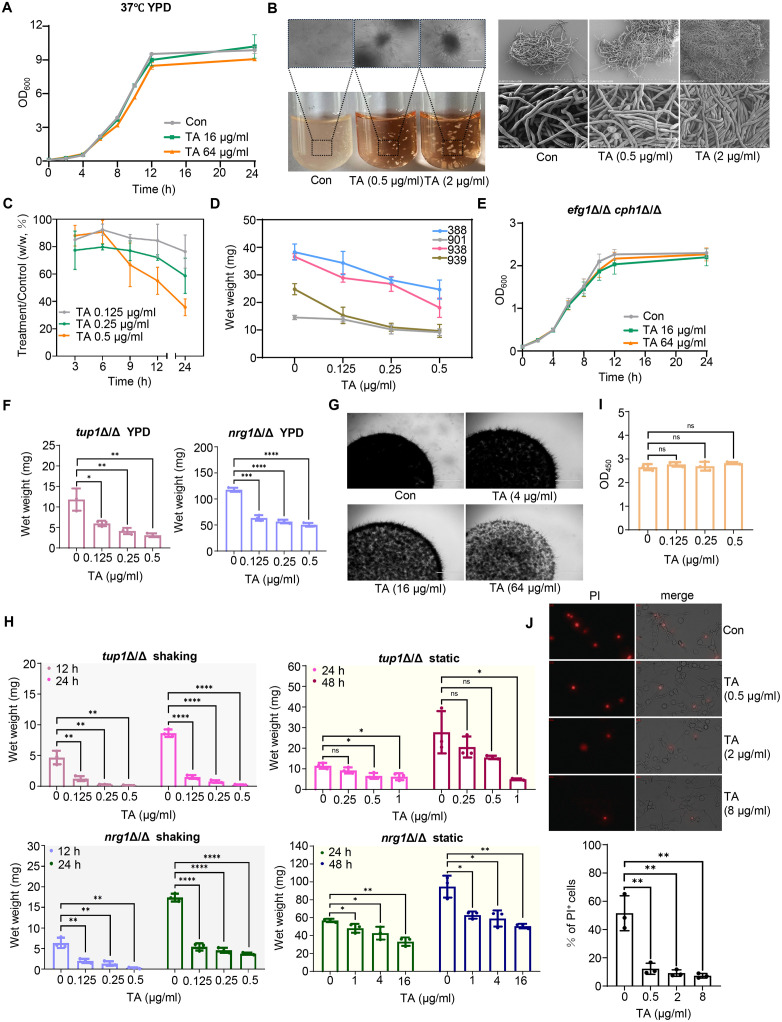
TA inhibits the proliferation of *C. albicans* hyphae but not yeast form. (A) Time-growth curves in *C. albicans* SC5314 (YPD, 37 °C). (B) Morphology of hyphal mass. *C. albicans* SC5314 were cultured in RPMI1640 and treated with ddH_2_O or TA at 37 °C for 24 h. Scale bars, top left and right, 100 μm, bottom right, 10 μm. (C) The wet weight ratio of *C. albicans* treated with TA and ddH_2_O (RPMI1640, 37 °C). (D) Wet weights of clinical isolated *C. albicans* 388, 901, 938, 939 in the presence of TA, 24 h). (E) The proliferation of yeast-blocked *efg1*Δ/Δ*cph1*Δ/Δ mutant (RPMI1640, 37 °C). (F) Wet weights of hyphae-blocked *tup1*Δ/Δ and *nrg1*Δ/Δ mutants (YPD, 37 °C). (G) Colonies of *C. albicans* growth on the RPMI1640 agar (37 °C, 24 h, scale bars, 1000 μm). (H) Wet weights of shaking or static *tup1*Δ/Δ and *nrg1*Δ/Δ mutants treated with TA (RPMI1640, 37 °C). (I) Metabolic activities assayed by CCK-8. *C. albicans* SC5314 was shaken in RPMI1640 at 37 °C with TA and equal wet weights of cells were assayed. (J) RAW 264.7 cells were co-incubated with *C. albicans* SC5314. *C. albicans* were treated with ddH_2_O or TA for 3 h, MOI = 0.5. Then, cells were stained with propidium iodide (PI). The percentage of PI-positive RAW 264.7 cells were calculated, Scale bars, 70 μm. Data were shown as mean ± SD, two-tailed unpaired t test (F, H, I, J), *p < 0.05, **p < 0.01, ***p < 0.001, ****p < 0.0001, ns, no significant.

To investigate the protective mechanism, the growth of *C. albicans* in the presence of TA were determined. Unexpectly, TA showed no inhibitory effect on the growth of *C. albicans* in YPD medium, even at a concentration of 64 μg/ml, which was 16 times of the MIC (Figs 2A and S1A). To rule out the impact of culture medium components on TA activity, fungal growth in RPMI1640 medium which was consistent with the medium used in MIC determination was tested. Interestingly, we observed that TA could promote aggregation of hyphae in RPMI1640 medium (Fig 2B). Scanning tunneling microscopy revealed that hyphae after the treatment of TA were regularly and tightly packed with minimal interstitial space between them (Fig 2B). Such dense cross-linkage restricted the proliferation of *C. albicans*, as tested by the wet weight of filtered cells. The wet weight of *C. albicans* was reduced dose-dependently over time by the treatment of TA (Fig 2C). Similar hyphal aggregates and inhibition with the treatment of TA were also observed in other *C. albicans* isolates (Figs 2D and S1B). These results suggest that TA promoted aggregation of hyphae in RPMI1640 medium at 37 °C and exerted significant inhibitory effects at low concentrations.

The weak inhibition of yeast form in YPD medium and significant inhibition of hyphae in RPMI1640 medium indicated that TA’s activity may be influenced by *C. albicans* morphology rather than nutrients. To test this hypothesis, we examined the sensitivity of *C. albicans* yeast-blocked *efg1*Δ/Δ*/cph1*Δ/Δ mutant and hyphae-blocked *tup1*Δ/Δ and *nrg1*Δ/Δ mutants to TA. The proliferation of *efg1*Δ/Δ*/cph1*Δ/Δ mutant was not inhibited by 64 μg/ml of TA in either RPMI1640 or YPD medium (Figs 2E and S1C). In contrast, the proliferation of hyphal *tup1*Δ/Δ and *nrg1*Δ/Δ mutants was inhibited by 0.125 μg/ml of TA in YPD medium (Fig 2F). Consistently, the proliferation of hyphae formed wide type *C. albicans* on solid RPMI1640 agar but not YPD agar was inhibited by TA, as the lighttight colonies on the RPMI1640 agar was less (Figs 2G and S1D). These results suggest that TA primarily targets *C. albicans* hyphae. To assess the necessity of space compression by aggregated hyphal clumps, TA’s activity was tested under the conditions of shaking and statics, as shaking promoted hyphal aggregation (S1E Fig). Our results showed that TA inhibited the proliferation of *tup1*Δ/Δ and *nrg1*Δ/Δ mutants in both shaking and static culture conditions. However, the MIC against these mutants was more increased in the standing static culture than in shaking condition, suggesting that the inhibitory effect of TA partially depended on hyphal aggregation (Fig 2H). The cell counting kit-8 (CCK-8) assay showed that TA-treated clumped hyphae had similar metabolic activities to the unclumped hyphae, indicating that the cells in the clumps were alive (Fig 2I). The co-incubation of *C. albicans* with murine monocyte-macrophage cells (RAW 246.7 cells) showed that TA treatment did not significantly affect the length of hyphae, but could reduce hyphal damage to cells (S1F Fig). Treatment with 0.5-8 μg/ml TA reduced the proportion of PI-positive dead cells by approximately 40% (Fig 2J). In addition, to evaluate the potential impact of TA on *C. albicans* in the mouse intestine, we stained the fecal matter with Calcofluor White (CFW). The results showed the presence of hyphae in the feces. Following TA treatment, large fluorescent aggregates were observed, but it was difficult to distinguish whether these were hyphal clumps (S1G Fig). Taken together, our results indicate that TA primarily inhibits *C. albicans* hyphal proliferation and its antifungal effect is enhanced by causing hyphal aggregation.

### TA promotes aggregation and inhibits proliferation of hyphae by targeting cell wall chitosan

**Fig 3:**
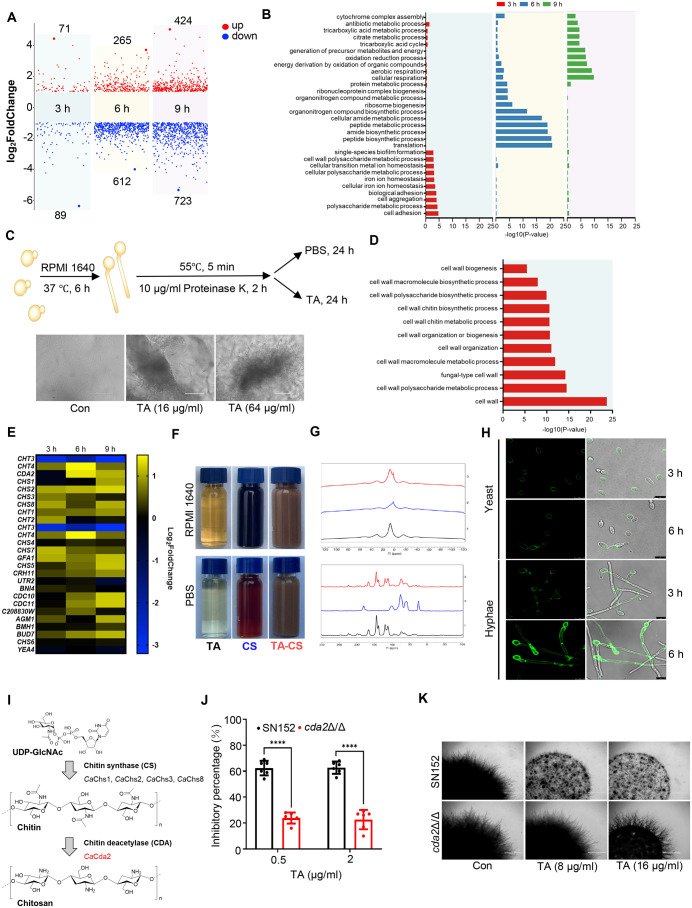
TA inhibits hyphal proliferation through cross-linking with chitosan. (A) Differentially expressed genes (DEGs) in *C. albicans* SC5314 cultured in RPMI1640 and treated with 2 μg/ml TA for 3, 6 and 9 h. (B) GO analysis of DEGs. (C) Schematic diagram and images of *C. albicans* SC5314 treated with Proteinase K. Scale bars, 100 μm. (D) GO analysis of cell wall enriched DEGs at 3 h. (E) Heatmap of chitin-associated DEGs in *C. albicans* treated with TA for 3 h. (F) Images of TA (30 mg/ml), CS (30 mg/ml) and flocculated TA-CS (Tannic acid-Chitosan oligosaccharide). (G) ^1^H (top) and ^13^C (bottom) solid-state NMR of TA (black), CS (blue) and TA-CS (red). (H) Eosin Y staining of yeast (YPD, 30 °C) and hyphae (RPMI1640, 37 °C) of *C. albicans* SC5314. (I) Chitin and chitosan synthesis in yeast. (J) Inhibitory percentages of TA against parental SN152, *cda2*Δ/Δ mutants. (K) Colonies of SN152 and *cda2*Δ/Δ mutants grown on RPMI1640 agar at 37 °C for 72 h (Scale bars, 1000 μm). Data were shown as mean ± SD, two-tailed unpaired t test (J), **p < 0.01, ****p < 0.0001.

Transcriptomics was used to investigate the antifungal mechanism of TA. With prolonged treatment of TA from 3 h to 9 h, the number of differentially expressed genes was increased from 160 to 1147 (Fig 3A). These changes might correlate with the observation that hyphal clumps were not apparent at 3 h but became evident at 6 h after TA treatment (S2A Fig). Gene ontology (GO) analysis revealed that at 3 h, the enriched processes were related to cell adhesion, cell wall polysaccharide metabolic process, and iron homeostasis. At 6 h and 9 h, the enriched processes mainly focused on protein metabolism and synthesis, oxidative respiration, and carbon metabolism (Fig 3B). These results indicated that TA mainly affected metabolism and proliferation after hyphal aggregation. Adhesions encoded by *ALS2*, *PGA13*, and *CSH1* were upregulated in TA-treated hyphae. Considering TA can bind non-specifically to many proteins, the inactivated hyphae treated with different agents were further assayed to explore the aggregations [16]. Our results showed that inactivation of hyphae with heat, alcohol, paraformaldehyde, or proteinase K did not change TA-mediated aggregation, preliminarily ruling out the possibility of direct binding of TA to adhesins or up-regulation of adhesins to promote hyphal aggregation (Figs 3C and S2B).

In addition to adhesions, genes involved in cell wall polysaccharide metabolism changed significantly in *C. albicans* treated with TA for 3 h. Among them, pathways related to chitin metabolism and biosynthesis were enriched (Fig 3D). Most of genes encoding chitin synthase (*CHS1*, *CHS2*, *CHS3*, *CHS5*, *CHS7*, *CHS8*), chitin deacetylases (*CDA2*), chitinase (*CHT1*, *CHT2*, *CHT4*) and genes regulating chitin metabolism, were up-regulated in *C. albicans* treated with TA (Fig 3E). Previously, several studies have found that TA can form coagulation-flocculation with chitosan (CS), a product of chitin deacetylation. Hydrogen bonds can be formed between the –OH of TA and the –OH and –NH_2_ groups of chitosan [17,18]. To investigate the potential flocculation, we mixed TA and chitosan at room temperature in RPMI1640 medium or PBS buffer. The mixture immediately induced flocculation and formed brown insoluble substances (TA-CS, insoluble in DMSO, DMF, ethyl acetate and acetone). The floccules were washed with ddH_2_O and characterized using solid-state nuclear magnetic resonance (NMR) spectroscopy and infrared spectrometer (IR). TA-CS exhibited separate characteristic peaks of TA and chitosan in terms of ^1^H NMR spectrum, ^13^C NMR spectrum and FTIR spectra, confirming the presence of both molecules in the floccules (Figs 3F and 3G, and S2C). To investigate whether the aggregation of hyphae is mediated by TA and chitosan, the presence of chitosan in *C. albicans* was detected by eosin Y, a dye that specifically binds to chitosan but not to chitin [19,20]. As shown in Fig 3H, the chitosan content in hyphae induced for 6 h was significantly higher than that in yeast form or in hyphae induced for 3 h, indicating the correlation between hyphal aggregation and chitosan.

Subsequently, mutants involved in chitosan synthesis were constructed to further confirm the target of TA. Gene homology analysis revealed that there is only one gene encoding chitin deacetylase, *CDA2*, in *C. albicans* (Fig 3I). However, the function of *CDA2* is inferred from its homologous sequences rather than being verified [21]. So, the *cda2*Δ/Δ mutants were constructed by CRISPR/Cas9 system (S2D Fig). As expected, the *cda2*Δ/Δ mutants were resistant to TA, which showed lower inhibitory percentage in comparison to the wild-type SN152. (Fig 3J). Consistently, the inhibitory effect of TA on parental SN152 was significantly higher than that on *cda2*Δ/Δ mutant on solid agar (Fig 3K). As expected, TA also failed to induce hyphal aggregation in *cda2*Δ/Δ mutant (S2E Fig). The interior of clumps in TA-treated hyphae of *cda2*Δ/Δ mutant was significantly looser than those in the parental strain (S2F Fig). Taken together, these results indicated that chitosan produced by Cda2 is the main target of TA.

### Cda2 promotes chitosan production and the proliferation in *C. albicans*

**Fig 4:**
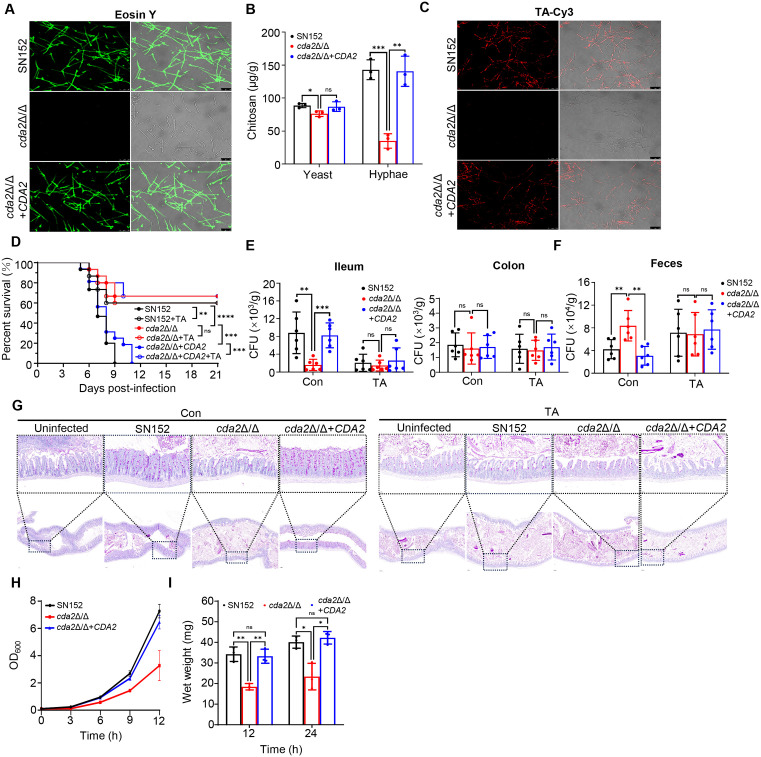
Chitin deacetylase *Cda2* is required for TA protection and *C. albicans* proliferation. (A) Eosin Y staining. *C. albicans* SN152, *cda2*Δ/Δ and *cda2*Δ/Δ + *CDA2* were cultured in RPMI1640 at 37 °C for 6 h. Scale bars, 25 μm. (B) ELISA quantification of chitosan in cell wall extracts from *C. albicans* SN152, *cda2*Δ/Δ and *cda2*Δ/Δ + *CDA2*. (C) Tannic acid-Cyanine3 (TA-Cy3) staining of *C. albicans* SN152, *cda2*Δ/Δ and *cda2*Δ/Δ + *CDA2* cultured in RPMI1640 at 37 °C for 6 h. Scale bars, 25 μm (D) Survival rates of mice infected with *C. albicans* SN152, *cda2*Δ/Δ and *cda2*Δ/Δ + *CDA2* (n = 15). Female ICR mice were treated with 20 mg/kg TA or not. (E) Fungal burden of ileum and colon (n = 6). (F) Fungal burden of feces (n = 6). (G) Periodic Acid-Schiff (PAS) staining of ileum. Scale bars, top, 50 μm, bottom, 200 μm. (H) Time-growth curves of *C. albicans* SN152, *cda2*Δ/Δ and *cda2*Δ/Δ + *CDA2* cultured in YPD media at 30 °C. (I) Wet weights of *C. albicans* SN152, *cda2*Δ/Δ and *cda2*Δ/Δ + *CDA2* cultured in RPMI1640 at 37 °C. Data were shown as mean ± SD (B, E, F), scale bars, 25 μm (A, C), two-tailed unpaired t test (B, E, F, I), Log-rank (Mantel-Cox) test (D), *p < 0.05, **p < 0.01, ***p < 0.001, ****p < 0.0001, ns, no significant.

Eosin Y staining showed that the content of chitosan in *cda2*Δ/Δ mutant was significantly decreased compared to the parental (SN152) and *CDA2* revertant (*cda2*Δ/Δ + *CDA2*) strains (Fig 4A). Similarly, a more specific ELISA assay also showed that the chitosan of cell wall extracts in *cda2*Δ/Δ mutant was significantly lower (Fig 4B). To confirm the binding of TA to *C. albicans* was chitosan-dependent, we synthesized a TA-Cy3 probe by the reaction of Cy3-COOH with -OH of TA (S3A Fig). TA-Cy3 was distributed on the hyphal surface of parental SN152 and *cda2*Δ/Δ + *CDA2* strain, but no fluorescence was observed in the *cda2*Δ/Δ mutant, indicating that TA binds mainly to the chitosan of cell wall (Fig 4C). In addition to diminishing the binding of TA to *C. albicans*, the disruption of *CDA2* abolished the protection of TA in the mouse model of gastrointestinal infection. As shown in Fig 4D, treatment with 20 mg/kg TA increased the survival rate of mice infected with SN152 and *cda2*Δ/Δ + *CDA2* strains by about 60%. Meanwhile, the virulence of *cda2*Δ/Δ mutant in the mouse gastrointestinal infection model was significantly reduced, and the mortality rate of infected mice was only about 66%. However, treatment with 20 mg/kg TA did not improve the survival of mice infected with *cda2*Δ/Δ mutant. Two days after infection, the fungal load in the ileum and colon was detected. Our results showed that TA treatment could effectively reduce the CFUs of the parental SN152 and *CDA2* revertant strain in the ileum. However, it had not reduced the number of the *cda2*Δ/Δ mutant. Consistently, fecal fungal load showed that TA treatment increased the CFUs of the SN152 and *CDA2* revertant strains, but failed to promote the excretion of the *cda2*Δ/Δ mutant (Fig 4E and 4F). PAS staining revealed that the intestinal oedema and inflammation were significantly reduced in mice infected with the *cda2*Δ/Δ mutant, or with the SN152 or *cda2*Δ/Δ + *CDA2* strains, when treated with TA (Fig 4G). These results suggest that *CDA2* is essential for maintaining *C. albicans* pathogenicity and the protection of TA is dependent on the cell wall chitosan. Given that the binding of TA and chitosan inhibited hyphal proliferation, we speculated that *CDA2* knockout would also reduce *C. albicans* proliferation. Compared with the parental SN152 and *CDA2* revertant strain (*cda2*Δ/Δ + *CDA2*), the proliferation of *cda2*Δ/Δ mutant was significantly slowed down in YPD or RPMI1640 medium, further strengthening the essential role of chitosan and *CDA2* in *C. albicans* growth (Figs 4H and 4I, and S3B and S3C).

### Chitosan disruption influenced the carboxylic acid metabolism and the abundance of cell wall proteins

**Fig 5:**
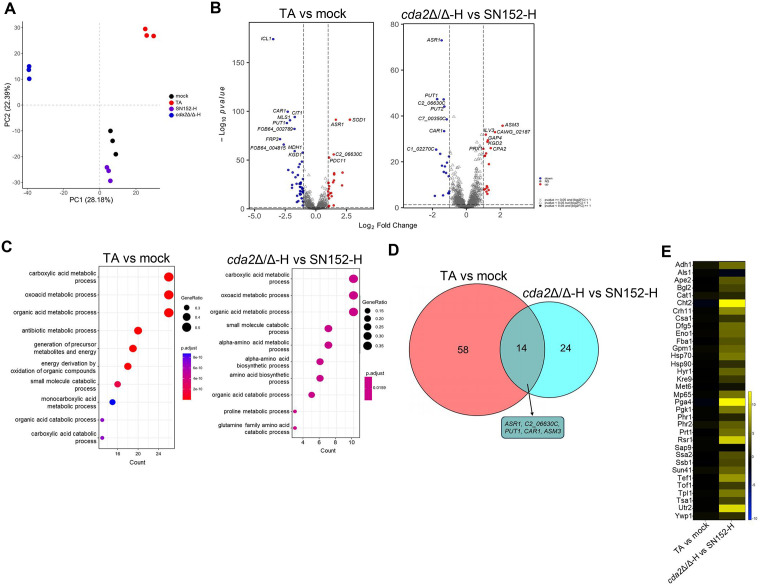
Analysis of differential proteins expressed in TA-treated *C. albicans* and the *cda2*Δ/Δ mutant. (A) Principal component analysis. Plots showed protein abundance in *C. albicans* SC5314 (mock, black), SC5314 treated with 2 μg/ml TA (TA, red), hyphae of *C. albicans* SN152 (SN152-H, purple) and hyphae of *cda2*Δ/Δ mutants (*cda2*Δ/Δ-H, blue). *C. albicans* were cultured in RPMI1640 at 37 °C for 12 h and each principal component (PC1 and PC2) shows the variance percentage. (B) Volcano plots of the differentially expressed proteins. (C) GO enrichment analysis of the differentially expressed proteins in TA-treated *C. albicans* and the *cda2*Δ/Δ mutant. (D) Venn diagram showing the overlap of differentially expressed proteins between TA-treated *C. albicans* and the *cda2*Δ/Δ mutant. (E) Heatmap showing the changes of fungal cell wall proteins in TA-treated *C. albicans* and the *cda2*Δ/Δ mutant.

TA significantly inhibited hyphal cell proliferation. Therefore, the different proteins expressed in hyphal cells were analyzed using Astral-DIA (data-independent acquisition) proteomics. A total of 2266 proteins were detected in the Orbitrap Astral and a clear stratification of the different treatments was observed using principal component analysis (PCA) (Fig 5A). As shown in Fig 5B, 72 differentially expressed proteins were detected in the hyphal cells treated with 2 μg/ml TA for 12 h, and 38 differentially expressed proteins were detected in the *cda2*Δ/Δ mutant hyphae compared to the parental SN152 hyphae. Gene ontology (GO) analysis showed that both TA treatment and *CDA2* knockout significantly altered the carboxylic acid metabolic process, oxoacid metabolic process and organic acid metabolic process. In addition, TA treatment could affect the energy generation process, which was associated with the inhibition of hyphal proliferation (Fig 5C). The Venn diagram showed that 14 of the differentially expressed proteins in the *cda2*Δ/Δ mutant were identical to those in the TA-treated hyphae (Fig 5D). The similarity of the differentially expressed proteins and enriched pathways further indicated that the target of TA was chitosan. Chitosan cross-linking or reduction may affect the abundance of cell wall proteins. Our analysis revealed that TA treatment did not alter the abundance of 34 detected fungal cell wall proteins. However, some cell wall proteins involved in polysaccharide metabolism were increased in the *cda2*Δ/Δ mutant, such as the chitinase Cht2, the cell wall transglycosylase Crh11 and the cell wall glycosidase Utr2. These changes may be related to the increased chitin levels discussed below (Fig 5E).

### Disruption of *CDA2* altered cell wall architecture, stress responses and invasion

**Fig 6:**
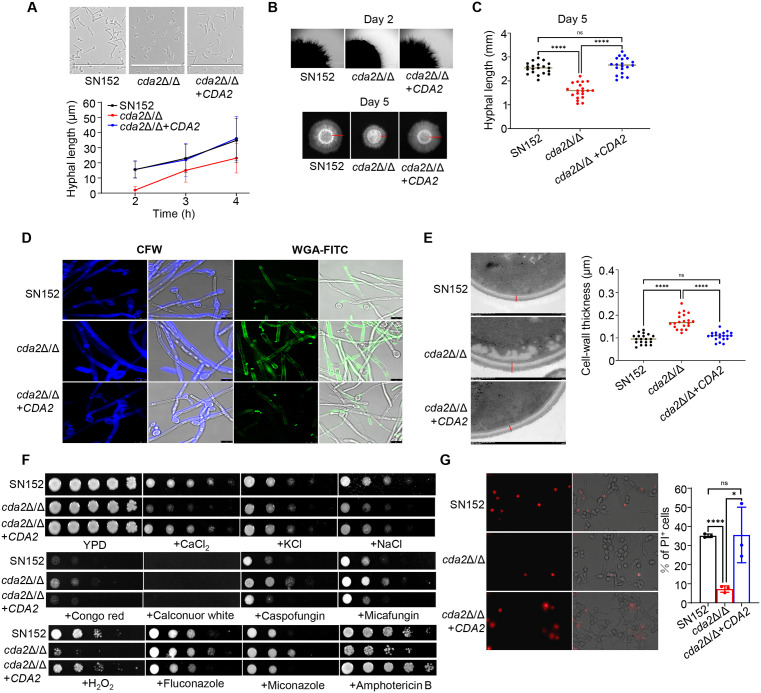
*Cda2* is required for the maintenance of *C. albicans* stress responses and virulence. (A) Hyphal germination and elongation. *C. albicans* SN152, *cda2*Δ/Δ and *cda2*Δ/Δ + *CDA2* were cultured in liquid RPMI1640 at 37 °C for 2 h,3 h and 4 h. The hyphal length was quantified by Image J. Scale bars, 100 μm. The hyphal length was measured by Image J (n = 40). (B) Hyphae colonies on RPMI1640 agar. *C. albicans* SN152, *cda2*Δ/Δ and *cda2*Δ/Δ + *CDA2* were grown on RPMI1640 agar at 37 °C for 2 days (top) and 5 days (bottom). Scale bars, top, 1000μm. (C) The hyphal length of *C. albicans* grown on RPMI1640 agar for 5 days was measured by Image J (n = 20). (D) Calcofluor white and wheat germ agglutinin staining. *C. albicans* SN152, *cda2*Δ/Δ and *cda2*Δ/Δ + *CDA2* were cultured in RPMI1640 at 37 °C for 6 h. Scale bars,7.5 μm. (E) Transmission electron microscope image of the cell wall. *C. albicans* SN152, *cda2*Δ/Δ and *cda2*Δ/Δ + *CDA2* were cultured in YPD at 30 °C for 9 h. Scale bars, 200 nm. Cell-wall thickness was measured by Image J (n = 20). (F) Spot assay. *C. albicans* were spotted on YPD agar containing CaCl_2_ (700 mM), KCl (2 M), NaCl (2 M), Congo red (500 μg/ml), calcofluor white (400 μg/ml), caspofungin (2 μg/ml), micafungin (2 μg/ml), H_2_O_2_ (8 mM), fluconazole (10 μg/ml), miconazole (3 μg/ml) and amphotericin B (0.3 μg/ml). (G) PI-positive RAW 264.7 cells co-incubated with *C. albicans*. *C. albicans* SN152, *cda2*Δ/Δ and *cda2*Δ/Δ + *CDA2* (MOI = 1) were incubated with RAW 264.7 cells for 3 h and stained with PI. Scale bars,70 μm. Data were shown as mean ± SD, two-tailed unpaired t test (C, E, G), *p < 0.05, ****p < 0.0001, ns, no significant.

The rigid and dynamic architecture and composition of the cell wall provide essential protection for fungal adaptation to an ever-changing environment [22]. The role of chitosan in the pathogenesis and stress response of *C. albicans* was therefore investigated. First, the hyphal budding of *cda2*Δ/Δ mutant was observed, as the cell wall polymers can influence the elasticity of the growing apices of the hyphae. As shown in Fig 6A, hyphal germination was delayed in the *cda2*Δ/Δ mutant, which germinated just after 2 h of induction at 37 °C. Meanwhile, the hyphal length was significantly shorter than that of the parent and the revertant mutant induced for the same time. Consistently, disruption of *CDA2* reduced hyphal growth on RPMI1640 solid agar (Fig 6B and 6C). As reported, high levels of chitin in the cell wall may cause *C. albicans* death [23]. We hypothesized that the delayed proliferation and hyphal germination of *cda2*Δ/Δ mutant were caused by chitin accumulation. CFW and FITC-conjugated wheat germ agglutinin (WGA-FITC) were used to detect the total and exposed chitin, respectively [24]. In the *cda2*Δ/Δ mutant, the fluorescence intensity of CFW and WGA-FITC was significantly enhanced in hyphae and slightly enhanced in yeast, indicating that the chitin content was increased (Figs 6D, and S4A and S4B). As well, the cell wall thickness of *cda2*Δ/Δ mutant was significantly increased, which was closely related with the elevated levels of chitin (Fig 6E). Secondly, the sensitivity of *C. albicans* to various stresses was tested. Our results showed that the disruption of *CDA2* could result in hypersensitity of *C. albicans* to osmotic stimulation (CaCl_2_, KCl, NaCl). Furthermore, *cda2*Δ/Δ mutants exhibited increased sensitivity to H_2_O_2_. Notably, the *cda2*Δ/Δ mutant with elevated chitin levels displayed enhanced resistance to azoles and echinocandins. These phenotypes align with previous studies that increased chitin content can enhance *C. albicans* resistance to both azoles and echinocandins [25–27]. In contrast, the disruption of *CDA2* did not result in significant changes in sensitivity to amphotericin B and the sensitivity of the *CDA2* revertant mutant to all stimuli was comparable to that of the parental strain SN152 (Fig 6F). These results highlight the importance of chitosan in modulating the cell wall responses. Finally, the co-incubation indicated that the *cda2*Δ/Δ mutant exhibited similarities to the TA-treated *C. albicans*, both of which were attenuated to invade the RAW 264.7 cells (Fig 6G). In summary, chitin deacetylation is crucial for regulating the proliferation, budding, stress responses and virulence of *C. albicans*.

## Discussion

*Candida* overgrowth in the gut is a major positive inducer of systemic infections [28]. Understanding the mechanism of *Candida* overgrowth is crucial for reducing the risk of systemic infection. Risk factors for invasive candidiasis in the ICU include the utilization of broad-spectrum antibiotics, immunosuppressive agents, total parenteral nutrition, and abdominal surgeries [29]. Additionally, gut dysmotility and dysbiosis of the intestinal microbiota are common in ICU patients and may also contribute to *Candida* overgrowth [30]. We hypothesized that the lack of vegetables and fruit may be a key factor leading to *Candida* overgrowth. This hypothesis was supported by our testing of the antifungal activity of ellagic acid, myricetin and tannic acid, which are commonly found in pomegranates, blueberries, bayberries, grapes, etc [31]. The average dietary intake of polyphenols is estimated to be around 1 g/day, with 90–95% being non-absorbable and mostly accumulated in the colon [10,32]. The volume of colon is about 0.4 L, so the polyphenols concentration could reach up to 2.5 mg/ml approximately [33]. The high concentration of polyphenols in the gut may be crucial for controlling the growth of *C. albicans*. In this study, we confirmed the protective effect of TA against gastrointestinal *C. albicans* infection, providing valuable insights for future research on the effects of polyphenols on fungal colonization and overgrowth in humans.

**Fig 7:**
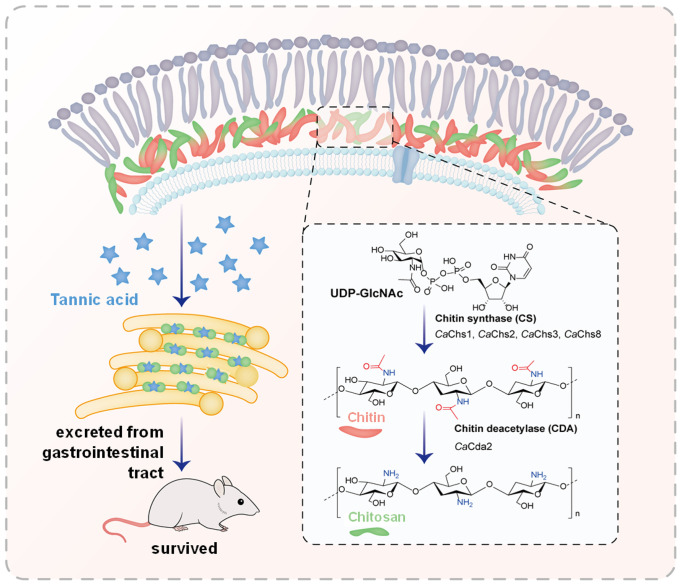
Tannic acid cross-links chitosan on the hyphal cell wall, and facilitates the excretion of hyphae from feces. The synthesis and deacetylation of chitin was shown on the right. Chitin and chitosan were colored as red and green.

Previous studies have reported that herbal tannins were effective against *C. albicans* and biofilms, but the specific mechanisms have not been elucidated [34,35]. Our study revealed that TA mainly acted on *C. albicans* hyphae and had no significant inhibitory effect on yeast. Although the effects of yeast and hyphae on *C. albicans* gut commensalism show inconsistent results in different mouse models, the role of hyphae in the secretion of virulence factors and tissue destruction is undeniable [36–38]. Recently, *Chang et al*. found that the peptide YY secreted by intestinal Paneth cells has the ability to eradicate *C. albicans* hyphae while preserving yeast commensalism, revealing a novel defense mechanism of the gut against virulent forms of *C. albicans* [39]. *C. albicans* in the gut is not only a risk for invasive candidiasis, its commensalism also has some potential benefits, such as inducing protective Th17 and antibody responses [4]. Therefore, elimination of hyphal *C. albicans* may be a less disruptive strategy to prevent candidemia. Our study showed that TA induced hyphal aggregation and promoted the excretion of hyphae from feces, which subsequently reduced *C. albicans* invasion (Fig 7). Using TA, an FDA-approved food additive, to prevent systemic *C. albicans* infection may have less impact on fungal homeostasis than using broad-spectrum antifungals.

The mechanism of TA inhibition on hyphae is unique and differs from the traditional idea that TA binds to proteins [40]. In our study, TA mainly bound to chitosan distributed on the surface of hyphae. The flocculation of TA and chitosan occurred in typical buffers, as confirmed by solid-state NMR and infrared spectroscopy. But due to the insolubility of floccules, the exact chemical structure was not identified. Cell wall chitosan is mainly derived from the deacetylation of chitin, but the amount of chitosan in different fungi varies greatly [12]. Many studies have demonstrated that chitosan is critical in fungal pathogenesis and immune evasion [41]. In *Cryptococcus neoformans* and *Cryptococcus gattii*, the loss of chitosan reduced their virulence and increased host immune recognition and inflammatory responses [42,43]. *Fusarium oxysporidium* and *Rivaromyces dahlia* can deacetylate chitin oligomers into chitosan to avoid recognition by chitin receptor complex in plant cells [41]. In contrast, the function of chitosan in *C. albicans* has not been studied. Our results showed that hyphal chitosan was greatly reduced in *cda2*Δ/Δ mutant. The reduction of chitosan in *cda2*Δ/Δ mutant resulted in a lack of TA binding targets, and thus the inhibition of TA on hyphae was significantly attenuated. During hyphal proliferation, chitin is usually enriched at the septum of *C. albicans,* and the chitin content in hyphae was significantly higher than that in yeast [44,45]. Our study showed that there was more chitosan in *C. albicans* hyphae. The proteomic analysis indicated that the interaction between TA and chitosan did not significantly alter the abundance of cell wall proteins. But it affects intracellular organic acid metabolism and the generation of energy. We have not detected all cell wall proteins in this study, primarily due to challenges associated with protein extraction and the sensitivity of mass spectrometry. A more specific approach for extracting cell wall proteins may provide more information. Combining the phenotypes of *cda2*Δ/Δ mutant, we speculated that TA binding may have an effect on the modification or degradation of chitosan, which in turn inhibits hyphal proliferation.

In summary, our study revealed that dietary plant-derived TA can inhibit the proliferation of hyphae, effectively eliminate *C. albicans* from the gastrointestinal tract. Dietary supplementation with polyphenols may be a potentially effective strategy for preventing systemic *C. albicans* infection in ICU patients. The antifungal activity of TA was mainly dependent on chitosan, highlighting the importance of chitin deacetylation in regulating cell wall dynamics.

## Materials and methods

### Ethics statement

All experiments involving animals were conducted according to the ethical policies and procedures approved by the Committee on Ethics of Medicine of Naval Medical University in Shanghai.

### Mouse model of gastrointestinal infection with *C. albicans*

The mouse model of *C. albicans* gastrointestinal infection was constructed as Murine *Candida albicans* GI Candidiasis (dietary approach) SOP with a few modifications (https://www.niaid.nih.gov/sites/default/files/sop_murinemodel_gi_disseminated_candidiasis_by_candida_albicans.pdf). 28–31 g female ICR mice were purchased from Shanghai Regan Biotechnology Co., Ltd. Female ICR mice were maintained on drinking autoclaved water containing 5% glucose and antibiotics (levofloxacin hydrochloride, LEV, 0.4 g/l) throughout the experiments. Mice were given an intraperitoneal injection of cyclophosphamide (CTX, 100 mg/kg) on two consecutive days before *C. albicans* infection. Next, the mice were orally administered 4 × 10^8^ CFU of *C. albicans*. Two hours later, the mice received an intraperitoneal injection of 100 mg/kg CTX, and were orally administered with tannic acid (TA) once a day. The mice were injected intraperitoneally with 100 mg/kg of CTX at 3 and 6 days after infection of *C. albicans*.

### Gut permeability test

The mouse model of *C. albicans* gastrointestinal infection was constructed. After infected with *C. albicans* or treated with 20 mg/kg of TA for 3 days, mice were fasted for 4 ~ 12 h prior to the measurements for avoiding the interferences of intestinal contents. Gut permeability was assessed with intragastric administration of 4-kDa FITC -Dextran (600 mg/kg). Blood was collected from the portal vein 4 h after intragastric administration of FITC-Dextran and centrifugated at 2500 rpm for 15 min to prepare plasm for measuring FITC-dextran concentration with a fluoresce microplate reader at the excitation wavelength of 490 nm and the emission wavelength of 520 nm. Standard curves for calculating the FITC-dextran concentration in the samples were prepared by diluting FITC-Dextran in blank plasm.

### Strain constructions

The transient CRISPR/Cas9 system was used to construct the *C. albicans CDA2* knockout mutant [46]. The *CAS9* gene was amplified by PCR using primers P7 and P8 from plasmid pV1093. And the single guide RNA (sgRNA) was amplified with primers P5 and P6 by annealing PCR, using the products of two separate PCRs containing the sgRNA sequence. Repair DNA was then amplified using primers CDA2-Re-F and CDA2-Re-R from plasmid pFA-HA-HIS1. PCR product was purified by using SanPrep Column PCR Product Purification Kit (Sangon Biotech, Shanghai). The *CAS9*, sgRNA and repair DNA PCR products were transformed into *C. albicans* parental strain SN152. The knockout mutants were selected on synthetic complete (SC) plates lacking histidine and confirmed by PCR using primers CDA2-Te-F and CDA2-Te-R.

To revert the phenotypes of *C. albicans cda2Δ/Δ* mutant, plasmid CIP10-ARG4-CDA2 was constructed [47]. To construct the CIP10-ARG4 plasmid, the *ARG4* gene fragment was amplified from plasmid pFA-HA-ARG4 by using primers ARG4-N1F and ARG4-N1R. Next, pCIP10 was digested by SacI and XbaI and ligated with *ARG4* gene fragment using pEASY-Basic seamless cloning and assembly kit (TransGen, Beijing). The CIP10-ARG4 *E. coli* strain was obtained by screening on lysogeny broth (LB) agar plates with ampicillin (100 μg/ml). The full-length *CDA2* was amplified from genomic DNA of *C. albicans* strain SN152 using primers CDA2-N1F and CDA2-N1R. And then, plasmid CIP10-ARG4 was digested by KpnI and ligated with *CDA2* gene fragment using pEASY-Basic Seamless Cloning and Assembly Kit to construct plasmid CIP10-ARG4-CDA2. After that, the pCIP10-ARG4-CDA2 was linearized by StuI and transformed into *C. albicans cda2*Δ/Δ mutant. The correct mutant was selected on SC plates lacking arginine and confirmed by genomic PCR using primers CDA2-T1F and CDA2-T1R.

### Lithium acetate-based transformation

The transformation of *C. albicans* was carried out as previously described with a few modifications [48]. *C. albicans* were cultured in 5 ml liquid YPD medium at 30 °C and 200 rpm overnight. Next, overnight cultures were diluted with 50 ml fresh YPD medium until reaching an OD_600_ of 0.2 and incubated at 30 °C and 200 rpm for 4 h until reaching an OD_600_ of 0.8. Single stranded DNA (ssDNA) was boiled at 100 °C for 10 min and immediately transfer to ice. When the suspensions reaching an OD_600_ of 0.8, *C. albicans* were collected by centrifuging at 2800 rpm for 5 min and resuspended in 5 ml sterile ddH_2_O. After washing for twice with ddH_2_O, *C. albicans* were gently resuspended in 500 μl LATE solution (0.1M LiAc in 1 × TE from Yeastmaker Yeast Transformation System 2 [Clontech, Beijing]). Afterwards, 100 μl of *C. albicans* suspensions, 3 μg of PCR products or other DNA fragments, 10 μl boiled ssDNA were mixed gently. Then, the reaction tubes were incubated at 30 °C for 30 min. After incubation, the reaction tubes were gently resuspended and added with 700 μl freshly made PLATE solution (8 ml 50% PEG, 1 ml 1 M lithium acetate and 1 ml 10 × TE from Yeastmaker Yeast Transformation System 2 [Clontech, Beijing]) mixing gently. The reaction tubes were incubated overnight at 30°C. On the second day, the reaction tubes were gently mixed and heat shocked at 44 °C for 15 min. *C. albicans* were collected by centrifuging at 5000 rpm for 1 min and washed with 1 ml sterile ddH_2_O twice. Finally, *C. albicans* were resuspended in 50 μl sterile ddH_2_O and transferred to selective solid agar.

### Determination of minimum inhibitory concentration (MIC)

To determine the minimum inhibitory concentration (MIC), *C. albicans* isolates were grown overnight in YPD medium at 30 °C, then washed with PBS and diluted 10^5^-fold in RPMI1640 medium. 0.1 ml of the diluted cultures were inoculated into a 96-well plate. A further 0.1 ml of diluted cultures was added to the first well and supplemented with dietary polyphenols. The drug-containing medium in the first well was then serially diluted 2-fold. After 48 h incubation at 30 °C, the MIC was determined as the lowest concentration of wells without visible growth of *C. albicans*.

### Fungal colony-forming units (CFUs) assay

The mouse model of gastrointestinal infection with *C. albicans* were described above. After infected or treated with 20 mg/kg of TA for 1, 2 and 4 days, the jejunum, ileum and colon were harvested. The jejunum, ileum and colon were fixed with 4% paraformaldehyde and stained with Periodic Acid-Schiff buffers. For CFU assay, the organs were mechanically homogenized in 1 ml (for jejunum) or 1.5 ml (for ileum and colon) sterile phosphate buffered saline (PBS). Feces of uninfected mice, *C. albicans* infected mice and infected mice treated with 20 mg/kg TA were harvested at 1-, 2-, 4-, 6- and 8-days post infection. The collected feces were then homogenized and resuspended in 1 ml sterile PBS. Fungal loads were determined by plating 10-fold serial dilutions of homogenates on Sabouraud Dextrose Agar (SDA) plates containing 50 μg/ml ampicillin and 15 μg/ml gentamicin and incubated at 30 °C for 48 h. Finally, the number of colonies was counted and normalized to the weights of organs or feces.

### Time-growth curves of *C. albicans*

To determine the time-growth curves, *C. albicans* were cultured overnight in YPD medium at 30 °C. The overnight activated cells were diluted with fresh YPD medium or RPMI1640 medium to OD_600_ = 0.1 in the presence of TA or ddH_2_O. The diluted cultures were inoculated at 30°C or 37°C and 180 rpm for 12 h or 24 h. OD_600_ was measured and recorded every 2 h, 3 h or 12 h. All time-growth curves were performed in at least 3 replicates.

### Imaging by microscopy, scanning electron microscopy or transmission electron microscopy

*C. albicans* were activated in YPD medium at 30 °C overnight. The overnight cultures were diluted 10^3^-fold with fresh RPMI1640 medium and added with TA or ddH_2_O. Then, 5 ml of diluted cultures were incubated in 50 ml sterile centrifuge tubes at 37 °C and 180 rpm for 24 h. Images of the *C. albicans* hyphal mass were then taken with a camera or microscope. To obtain images of *C. albicans* hyphal mass by scanning electron microscope, overnight cultures were diluted 10^2^-fold with fresh RPMI1640 medium in the presence of TA or ddH_2_O. And 5 ml diluted cultures in 50 ml sterile centrifuge tubes were incubated at 37 °C and 180 rpm for 6 h. After incubation, images of the *C. albicans* were taken using HITACHI Regulus 8100 or HITACHI HT7800. To measure cell-wall thickness, transmission electron microscope images showing the cell wall of *C. albicans* were chosen and quantified using Image J.

### Inactivation of *C. albicans* hyphae

Overnight activated *C. albicans* were diluted 10^2^-fold with fresh RPMI1640 medium. And 5 ml of diluted cultures in 50 ml sterile centrifuge tubes were incubated at 37 °C and 180 rpm for 6 h. After incubation, the cultures were centrifuged at 4000 rpm for 5 min. *C. albicans* were collected and washed three times with sterile PBS (4000 rpm, 5 min). Then, cells were treated with 55 °C for 5 min (after heated inactivation, 100 μg/ml proteinase K was added and incubated at 37 °C for 2 h in PBS), 75% alcohol for 10 min or 4% paraformaldehyde for 10 min. *C. albicans* were then resuspended with 5 ml sterile PBS in the presence of TA (16 μg/ml, 64 μg/ml) or ddH_2_O and incubated at 37 °C and 180 rpm for 24 h. Images of the *C. albicans* hyphal mass were taken under a microscope.

### Determination of the wet weight

Overnight activated *C. albicans* were diluted 10^3^-fold with fresh liquid YPD medium or RPMI1640 medium in the presence of TA or ddH_2_O. 5 ml of diluted cultures in 50 ml sterile centrifugal tubes were incubated at 30 °C or 37 °C and 180 rpm for 12 h or 24 h. The *C. albicans* was collected using vacuum pump filters. Next, the wet weights of *C. albicans* were measured by using standard laboratory balances. The inhibitory percentages of TA against *C. albicans* were normalized to cells treated with ddH_2_O. The wet weight ratio between SC5314 treated with TA and SC5314 treated with ddH_2_O (control) was calculated.

### Spot assay

*C. albicans* SC5314, SN152 and *cda2*Δ/Δ single colonies were incubated overnight in YPD medium at 30 °C and 180 rpm. On the second day, the overnight cultures were diluted with sterile PBS (OD_600_ = 0.1). And, 1 μl of diluted fungal suspensions were spotted on YPD agar or RPMI1640 agar in the presence of TA or ddH_2_O. The agar was incubated at 30 °C or 37 °C for 24–72 h. Finally, images were taken with a camera or microscope.

*C. albicans* SN152, *cda2*Δ/Δ and *cda2*Δ/Δ + *CDA2* mutant were incubated in YPD medium overnight, and the overnight cultures were diluted with YPD medium (OD_600_ = 0.4). Subsequently, fungal suspensions were serial five-fold diluted in YPD to five concentration gradients. Then, 2 µl of diluted fungal suspensions were spotted on YPD agar containing CaCl_2_ (700 mM), KCl (2 M), NaCl (2 M), Congo red (500 μg/ml), calcofluor white (400 μg/ml), caspofungin (2 μg/ml), micafungin (2 μg/ml), H_2_O_2_ (8 mM), fluconazole (10 μg/ml), miconazole (3 μg/ml) and amphotericin B (0.3 μg/ml). All these agars were incubated at 30 °C for 48 h and photographed.

### Metabolic assay

*C. albicans* was cultured overnight in YPD medium at 30 °C and 180 rpm. The overnight cultures were then diluted 10^3^-fold with fresh or RPMI1640 medium in the presence of TA or ddH_2_O. Then 5 ml of diluted cultures were incubated in 50 ml sterile centrifuge tubes at 37 °C and 180 rpm for 24 h. After incubation, *C. albicans* was collected using vacuum pump filters. The wet weights of *C. albicans* were measured and 1 mg of *C. albicans* was resuspended with 2 ml sterile PBS in the presence of 220 μl CCK-8 solutions (TargetMol Chemicals Inc.). *C. albicans* was incubated at 37 °C for 2 h. Finally, the cells were centrifuged at 4000 rpm for 5 min and the OD_450_ of supernatants was measured using a microplate reader.

### Calcofluor white (CFW) staining of *C. albicans* in mouse feces

After infected with *C. albicans* or treated with 20 mg/kg of TA for 1 and 2 days according to the mouse model of gastrointestinal infection with *C. albicans*, the feces were harvested respectively. Next, the feces were added with 1.5 ml PBS and soaked overnight. 1.5 ml 10% KOH solution were then added in the feces and mixed. After incubated for 5 min, the feces were washed with 1 ml PBS, and added with 30 µg/ml calcofluor white incubated for 15 min at 4 °C without light. Excess dye was washed three times with 1 ml PBS and resuspended in 100 µl PBS. The fluorescence of calcofluor white was observed at an excitation wavelength of 355 nm and an emission wavelength of 440 nm using Olympus CKX53 inverted microscope.

### RNA sequence

*C. albicans* SC5314 was activated in YPD medium at 30 °C and 180 rpm overnight. Then cells were diluted in RPMI1640 medium to OD_600_ = 0.4 and treated with 2 μg/ml of TA for 3 h, 6 h and 9 h at 37 °C. Cells were washed and the total RNA was extracted using KALANG Fungal RNA Column extraction kit (KALANG, China). After the qualifying the RNA concentrations, mRNA was enriched and interrupted into length of 200–300 base pairs. The first and second strands of cDNA were then synthesized and purified. The purified double-stranded cDNA was end-repaired, ligated to A-tailed cDNA fragments and linked to sequencing adaptors, followed by fragment size selection, and finally PCR enrichment to obtain the final cDNA library. For the transcriptomic analysis, short sequences (reads) were mapped to the reference sequences (https://www.ncbi.nlm.nih.gov/genome/21?genome_assembly_id=294796) using HISAT2, and the number of reads of each gene was used to estimate the level of gene expressions. The DEGs were annotated by P value < 0.05 and absolute log_2_(fold change) ≥ 1. The GO enrichment analysis of DEGs was performed using Fisher’s exact test to evaluate the significance level of protein enrichment for a certain GO function entry. Functional enrichment analysis of differentially expressed genes is performed in GO functional entries, and the results allow a visual representation of the overall functional enrichment characteristics of all differentially expressed genes.

### Flocculation and characterization of TA and chitosan

90 mg of TA or chitosan oligosaccharide (CS) was dissolved in 3 ml of RPMI1640 medium or sterile PBS. Then, 1 ml of 30mg/ml TA and 1ml of 30 mg/ml CS was mixed to form tannic acid-chitosan oligosaccharide (TA-CS). Images of TA, CS and TA-CS solution were taken with a camera. For solid-state NMR and IR spectroscopy, 10 ml of 30 mg/ml TA and 10 ml of 30 mg/ml CS solution were mixed together, and then incubated (37 °C, 180 rpm) in a 50 ml sterile centrifuge tube for 1 h. After incubation, the precipitation was collected using vacuum pump filters and washed five times with sterile ddH_2_O and dried at 50 °C. After drying, 0.6 g TA, 0.6 g CS and 0.6 g TA-CS were tested by Bruker Avance Neo 400WB and Thermo Scientific Nicolet iS20.

### Eosin Y staining

The Eosin Y staining of *C. albicans* was carried out as previously described with a few modifications [20]. *C. albicans* was cultured in YPD medium at 30 °C overnight and diluted 10-fold with fresh liquid YPD medium or RPMI1640 medium. The diluted cultures were then incubated at 30 °C (yeast, YPD medium) or 37 °C (hyphae, RPMI1640 medium) and 180 rpm for 3 h or 6 h. After incubation, *C. albicans* were collected and washed twice with 1 ml McIlvaine’s buffer (0.2 M Na_2_HPO_4_ and 0.1 M citric acid [pH 6.0]). *C. albicans* were then resuspended in 500 μl McIlvaine’s buffer and added with 30 μl of eosin Y stocks (5 mg/ml, dissolved in ethanol absolute). *C. albicans* were incubated for 10 min at room temperature without light. Excess dye was washed twice with 1 mL of McIlvaine’s buffer and resuspended in 500 μl of McIlvaine’s buffer. The fluorescence of eosin Y was observed at an excitation wavelength of 488 nm and an emission wavelength of 500–650 nm using Leica Stellaris 8 STED scanning microscope.

### Synthesis of tannic acid-cyanine3 (TA-Cy3)

10 mg of CY3-COOH was dissolved in 3 ml of N, N-dimethylformamide (DMF). TA (1.2 eq), N, N’-diisopropylcarbodiimide (DIC, 100 eq), 1-hydroxybenzotriazole (HOBt, 1 eq) and 4-dimethylaminopyridine (DMAP, 1 eq) were added to the reaction tube and completely dissolved. The mixture was stirred at room temperature for 24 h under a nitrogen blanket. The solvent was evaporated under reduced pressure by rotary evaporation and large amounts of acetone were poured into the reaction tube. The reaction product was then filtered and collected. Tannic acid-cyanine3 (TA-Cy3) was obtained by vacuum drying. The fluorescence properties of TA-Cy3 were determined using a fluorescence spectrophotometer.

### TA-Cy3 staining

*C. albicans* SN152 and *cda2*Δ/Δ were cultured in YPD medium at 30 °C overnight. Next, overnight cultures were diluted 10-fold with fresh liquid YPD medium or RPMI1640 medium. 5 ml of diluted cultures were added with TA-Cy3 (0.5 μg/ml) or ddH_2_O in 50 ml sterile centrifuge tubes and incubated at 37 °C and 180 rpm for 6 h. After that, *C. albicans* cells were collected and washed three times with 1 ml sterile PBS. Finally, the *C. albicans* were resuspended in 0.5 ml sterile PBS and examined with Leica Stellaris 8 STED scanning microscope.

### Chitosan extraction and quantification

*C. albicans* SC5314 and *cda2*Δ/Δ were grown in YPD media at 30 °C overnight and then diluted in YPD and RPMI1640 medium to an OD_600_ of 0.2, respectively. Cells were grown at 30°C for 6 h and then centrifuged. The wet weight of *C. albicans* was determined and 50 ml of 1 M NaOH was added. Samples were incubated at 100 °C for 2 h with constant shaking. Cells were washed 5 times with ddH_2_O and centrifuged at 5000 rpm for 10 min. The residue was resuspended with 10% acetic acid and incubated in a 100 °C water bath for 3 h with constant shaking. The pH of the samples was adjusted to 7.0 with solid NaOH, then the suspension was centrifuged at 13000 × g for 10 min. The supernatant was removed for ELISA detection. Chitosan quantification was determined using the chitosan assay kit (Abcam) according to manufacturer’s instructions. The concentration of chitosan is calculated from the standard curves.

### Co-incubation of RAW 264.7 cells and *C. albicans*

The RAW 264.7 cells were cultured in DMEM medium supplemented with 10% FBS, 100 U/ml penicillin, and 10 μg/ml streptomycin in a humidified 5% CO_2_ incubator at 37 °C. 2 × 10^6^ of RAW 264.7 cells were added to a 6-well plate with 2 ml of DMEM medium. Then, 10 μl of *C. albicans* SC5314 (1 × 10^8^ CFU/ml), SN152 (2 × 10^8^ CFU/ml), *cda2*Δ/Δ (2 × 10^8^ CFU/ml) and *cda2*Δ/Δ + *CDA2* (2 × 10^8^ CFU/ml) were added to each well respectively. Additionally, RAW 264.7 cells co-cultured with *C. albican* SC5314 in the presence of TA or ddH_2_O.And these plates were placed at 37 °C in a humidified 5% CO_2_ incubator for 3 h, stained with 200 ng/ml propidium iodide (PI, Meilunbio, MA0137), and incubated at 37 °C for 15 min without light. These plates were photographed at an excitation wavelength of 535 nm and an emission wavelength of 615 nm using an olympus ckx53 microscope. The percentage of PI-positive RAW 264.7 cells was calculated by the number of red fluorescent dots.

### *C. albicans* hyphae imaging

*C. albicans* SN152, *cda2*Δ/Δ and *cda2*Δ/Δ + *CDA2* single colonies were incubated overnight in YPD medium at 30 °C and 180 rpm. Next, the overnight cultures were diluted 10^3^-fold with RPMI1640 medium. The diluted cultures were then incubated at 37 °C for 2 h, 3 h and 4 h. After incubation, images were taken with a microscope and the hyphal length was measured by Image J.

### Calcofluor white (CFW) staining

*C. albicans* was cultured in YPD at 30 °C overnight and diluted 10-fold with fresh liquid YPD medium or RPMI1640 medium incubated at 30 °C (yeast, YPD medium) or 37 °C (hyphae, RPMI1640 medium) and 180 rpm for 6 h. After that, *C. albicans* were collected and washed three times with 1 ml PBS. *C. albicans* were then resuspended in 1 ml PBS and added with 30 µg/ml calcofluor white, incubated for 15 min at 4 °C without light. Excess dye was washed three times with 1 ml PBS and resuspended in 100 µl PBS. The fluorescence of calcofluor white was observed at an excitation wavelength of 355 nm and an emission wavelength of 440 nm using a Leica SP5 scanning confocal microscopy.

### Wheat germ agglutinin-Fluorescein (WGA-FITC) staining

*C. albicans* was cultured in YPD at 30 °C overnight and diluted 10-fold with fresh liquid YPD medium or RPMI1640 medium incubated at 30 °C (yeast, YPD medium) or 37 °C (hyphae, RPMI1640 medium) and 180 rpm for 6 h. Then, *C. albicans* were washed three times with 1 ml PBS and resuspended in 4% paraformaldehyde. After an hour, *C. albicans* were washed three times and resuspended with 1 ml PBS. Subsequently, *C. albicans* were added with 100 µg/ml Wheat germ agglutinin-Fluorescein, incubated for 1 h. Excess dye was washed three times with 1 ml PBS and resuspended in 100 µl PBS. The fluorescence of wheat germ agglutinin was observed at an excitation wavelength of 490 nm and an emission wavelength of 525 nm using a Leica SP5 scanning confocal microscopy.

### Astral-DIA proteomics

*C. albicans* SC5314, SN152 and *cda2*Δ/Δ was activated in YPD medium at 30 °C and 180 rpm overnight. *C. albicans* SC5314 was diluted 10^3^-fold with RPMI1640 medium incubated at 37 °C and 180 rpm treated with ddH_2_O or 2 µg/ml of TA for 12 h. And *C. albicans* SN152 and *cda2*Δ/Δ were was diluted 10^3^-fold with RPMI1640 medium incubated at 37 °C and 180 rpm for 12 h. Next, cells were washed three times with PBS and collected at 3000–5000 g. Then, the collected cells were quick-frozen with liquid nitrogen. Three replicates of the samples are prepared to be stored at -80 °C. After that, the protein of *C. albicans* was extracted, quantified, detected, digested and desalted, fraction separated and detected by mass spectrometry. For the proteome analysis, we first performed principal component analysis of protein quantification values on all samples, and each principal component (PC1 and PC2) shows the variance percentage. And then, The DESeq2 based on negative binomial distribution were used to analysis protein expression abundance, and specifically used R language scripts based on R packages such as BiocManager, getopt, ggplot2, DESeq2, etc. Pairwise comparisons were performed between individual sample groups to find proteins that were differently expressed in different groups. The screening criteria for differential expressed proteins is log 2 (FoldChange)> 1 and p-value < 0.05. All differential proteins were mapped to the individual terms of the Gene Ontology database (https://www.geneontology.org/). Meanwhile, the number of proteins per term was counted and hypergeometric test was applied to identify GO entries that were significantly enriched in the differential proteins compared to all protein backgrounds.

### Quantification and statistical analysis

Statistical analyses were performed using the analysis of two-tailed unpaired t test and Log-rank (Mantel-Cox) test through GraphPad Prism 9 software (GraphPad Software). All data were represented as means ± SD, and were statistically considered when p values < 0.05 by statistical analysis methods, including data of animal experimentation and experiments *in vitro*. * p < 0.05, ** p < 0.01, *** p < 0.001, **** p < 0.0001.

## Supporting information

S1 Fig TA inhibits the proliferation of *C. albicans* hyphae, related to Fig 2. (A)Time-growth curves of *C. albicans* SC5314 in YPD medium at 30 °C. (B) Morphology of hyphal mass. Clinical isolated *C. albicans* 388, 901, 938, 939 were cultured in RPMI1640 and treated with double-distilled water or TA at 37 °C for 24 h. Scale bars, 200 μm. (C) The proliferation of yeast-blocked *efg1*Δ/Δ*cph1*Δ/Δ mutants in YPD medium at 37 °C. (D) Colonies of *C. albicans* SC5314 grown on YPD agar at 30 °C for 48 h. (E) Morphology of hyphae. *C. albicans* SC5314 was cultured in RPMI1640 and treated with double-distilled water or TA at 37 °C for 24 h or 48 h without shaking. Scale bars, 400 μm. (F) *C. albicans* SC5314 were cultured in liquid RPMI1640 and treated with double-distilled water or TA at 37 °C for 1 h,2 h and 3 h. The hyphal length was quantified by Image J. Scale bars, 100 μm. The hyphal length was measured by Image J (n = 40). (G) Calcofluor white staining of *C. albicans* in mouse feces. Data were shown as mean ± SD (A, C, F).
(TIF)

S2 Fig TA induces hyphal aggregation through cross-linking with chitosan, related to Fig 3. (A) Morphology of hyphal mass. *C. albicans* SC5314 was cultured in RPMI1640 and treated with ddH_2_O or TA at 37 °C for 3, 6 and 9 h. Scale bars, 400 μm. (B) Morphology of hyphal mass. *C. albicans* SC5314 was cultured in RPMI1640 at 37°C for 6 h and treated with 55 °C, 75% alcohol and 4% paraformaldehyde, then cultured in PBS and treated with ddH_2_O or TA at 37 °C for 24 h. Scale bars, 100 μm. (C) FTIR spectra of TA (black), CS (blue) and TA-CS (red). (D) Schematic diagram of the construction of knockout and revertant strains using CRISPR/Cas9. (E) Confirmation of the construction of and *cda2*Δ/Δ mutants identified by genomic PCR, compared to SN152 (parental, WT). (F) Morphology of hyphal mass. *C. albicans* SN152 and *cda2*Δ/Δ mutants cultured in RPMI1640 were treated with ddH_2_O or TA at 37 °C for 24 h. Scale bars,100 μm. (G) Scanning electron microscope image of the hyphal mass. *C. albicans* SN152 and *cda2*Δ/Δ mutants were cultured in RPMI1640 and treated with ddH_2_O or TA at 37 °C for 6 h. Scale bars,10 μm.
(TIF)

S3 Fig Chitin deacetylase *Cda2* is required for the inhibitory effect of TA on hyphae, related to Fig 4. (A) Chemical structures of TA-Cy3. (B) Confirmation of the construction of *CDA2* revertant mutant (*cda2*Δ/Δ + *CDA2*) identified by PCR. (C) Inhibitory percentage of TA against *C. albicans* SN152, *cda2*Δ/Δ and *cda2*Δ/Δ + *CDA*2. Data were shown as mean ± SD, two-tailed unpaired t test, *p < 0.05, **p < 0.01, ns, no significant.
(TIF)

S4 Fig The Chitin content measured by CFW and WGA-FITC staining, related to Fig 6. (A) Calcofluor white staining. (B)Wheat germ agglutinin staining. Yeast cells of *C. albicans* SN152, *cda2*Δ/Δ and *cda2*Δ/Δ + *CDA2* were cultured in YPD at 30 °C for 6 h. Scale bars,7.5 μm.
(TIF)

S1 Table Key sources table. (DOCX)

S1 Data Data that underlies this paper. Table containing the underlying data for Figs 1B, 1C, 1F, 1H, 1I, 2A, 2C, 2D–F, 2H–J, 3J, 4B, 4D–F, 4H, 4I, 6A, 6C, 6E, 6G, S1A, S1C, S1F and S3C.
(DOCX)

S2 Data Data that underlies this paper. Excel spreadsheet containing the underlying data for Figs 3A, 3B, 3D, 3E, 3G and S2C.
(XLSX)

S3 Data Data that underlies this paper. Excel spreadsheet containing the underlying data for Fig 5A–E.
(XLSX)
