# Supplementary figures and images for: Dietary tannic acid promotes intestinal clearance of C. albicans by cross-linking hyphal chitosan
Source: PLoS Pathog. 2025 Oct 15;21(10):e1013596. doi: 10.1371/journal.ppat.1013596 (PMC12543286; doi:10.1371/journal.ppat.1013596)

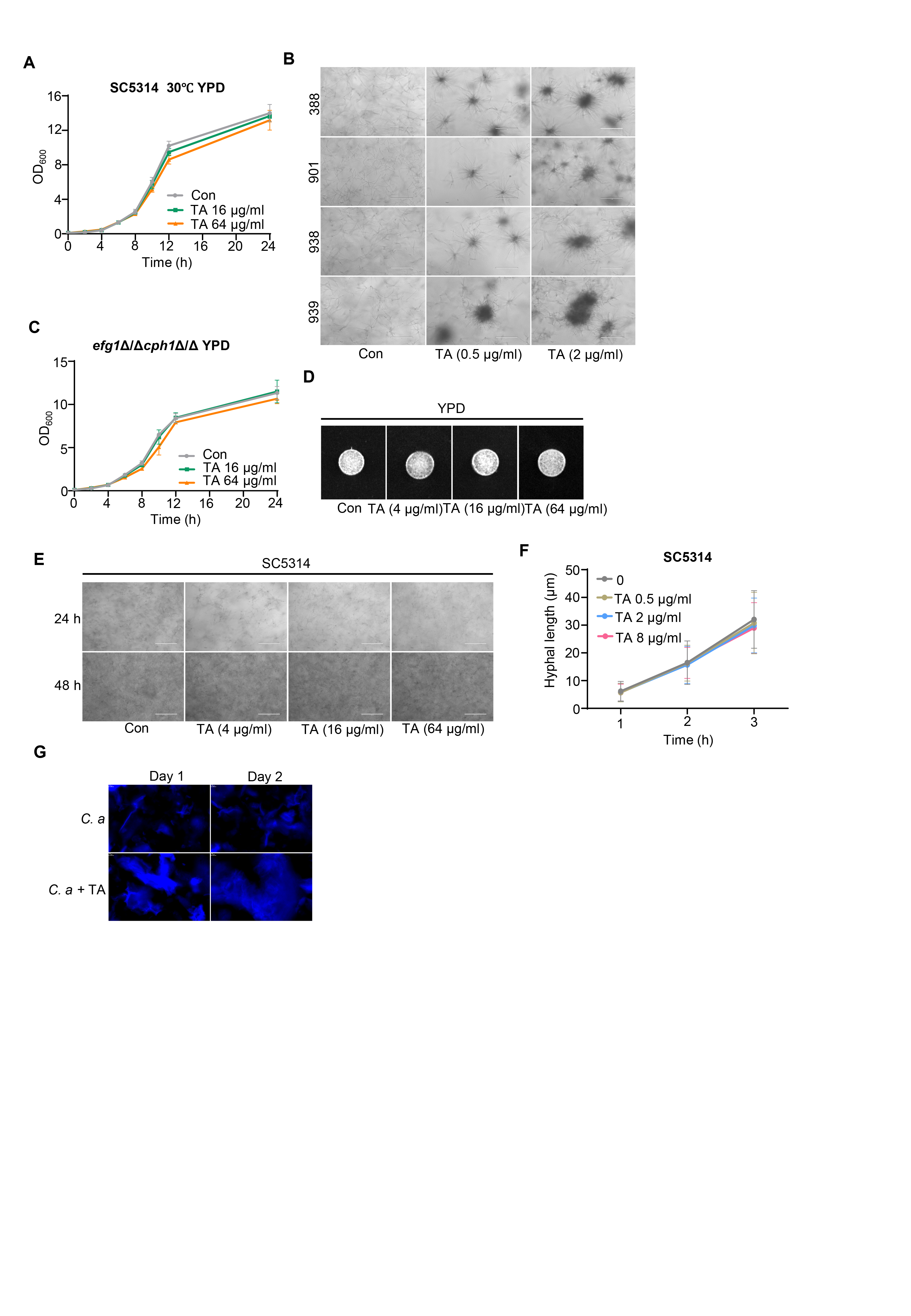

Supplement: S1 Fig — (A)Time-growth curves of C. albicans SC5314 in YPD medium at 30 °C. (B) Morphology of hyphal mass. Clinical isolated C. albicans 388, 901, 938, 939 were cultured in RPMI1640 and treated with double-distilled water or TA at 37 °C for 24 h. Scale bars, 200 μm. (C) The proliferation of yeast-blocked efg1Δ/Δcph1Δ/Δ mutants in YPD medium at 37 °C. (D) Colonies of C. albicans SC5314 grown on YPD agar at 30 °C for 48 h. (E) Morphology of hyphae. C. albicans SC5314 was cultured in RPMI1640 and treated with double-distilled water or TA at 37 °C for 24 h or 48 h without shaking. Scale bars, 400 μm. (F) C. albicans SC5314 were cultured in liquid RPMI1640 and treated with double-distilled water or TA at 37 °C for 1 h,2 h and 3 h. The hyphal length was quantified by Image J. Scale bars, 100 μm. The hyphal length was measured by Image J (n = 40). (G) Calcofluor white staining of C. albicans in mouse feces. Data were shown as mean ± SD (A, C, F). (TIF) [file ppat.1013596.s001.tif]

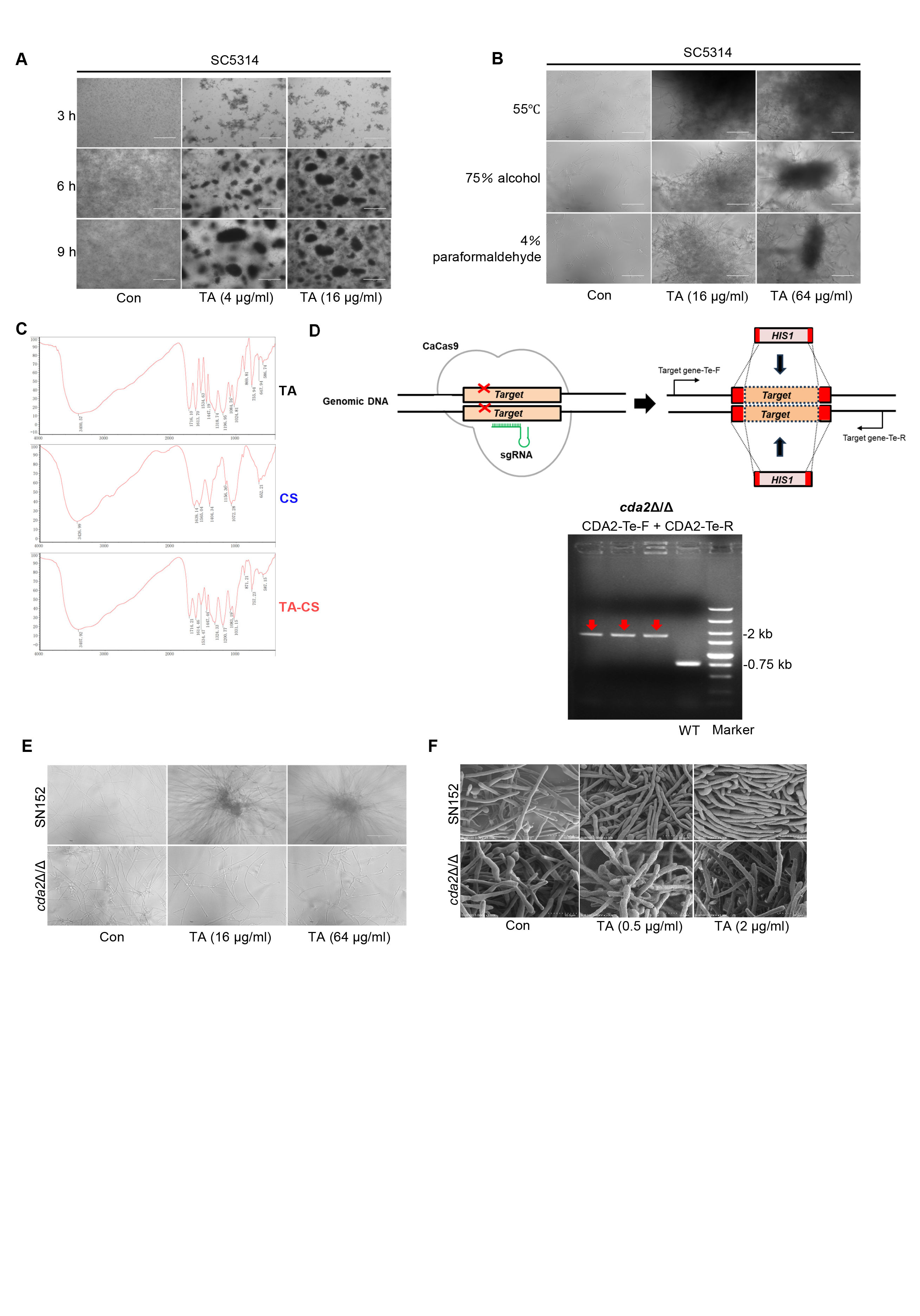

Supplement: S2 Fig — (A) Morphology of hyphal mass. C. albicans SC5314 was cultured in RPMI1640 and treated with ddH2O or TA at 37 °C for 3, 6 and 9 h. Scale bars, 400 μm. (B) Morphology of hyphal mass. C. albicans SC5314 was cultured in RPMI1640 at 37°C for 6 h and treated with 55 °C, 75% alcohol and 4% paraformaldehyde, then cultured in PBS and treated with ddH2O or TA at 37 °C for 24 h. Scale bars, 100 μm. (C) FTIR spectra of TA (black), CS (blue) and TA-CS (red). (D) Schematic diagram of the construction of knockout and revertant strains using CRISPR/Cas9. (E) Confirmation of the construction of and cda2Δ/Δ mutants identified by genomic PCR, compared to SN152 (parental, WT). (F) Morphology of hyphal mass. C. albicans SN152 and cda2Δ/Δ mutants cultured in RPMI1640 were treated with ddH2O or TA at 37 °C for 24 h. Scale bars,100 μm. (G) Scanning electron microscope image of the hyphal mass. C. albicans SN152 and cda2Δ/Δ mutants were cultured in RPMI1640 and treated with ddH2O or TA at 37 °C for 6 h. Scale bars,10 μm. (TIF) [file ppat.1013596.s002.tif]

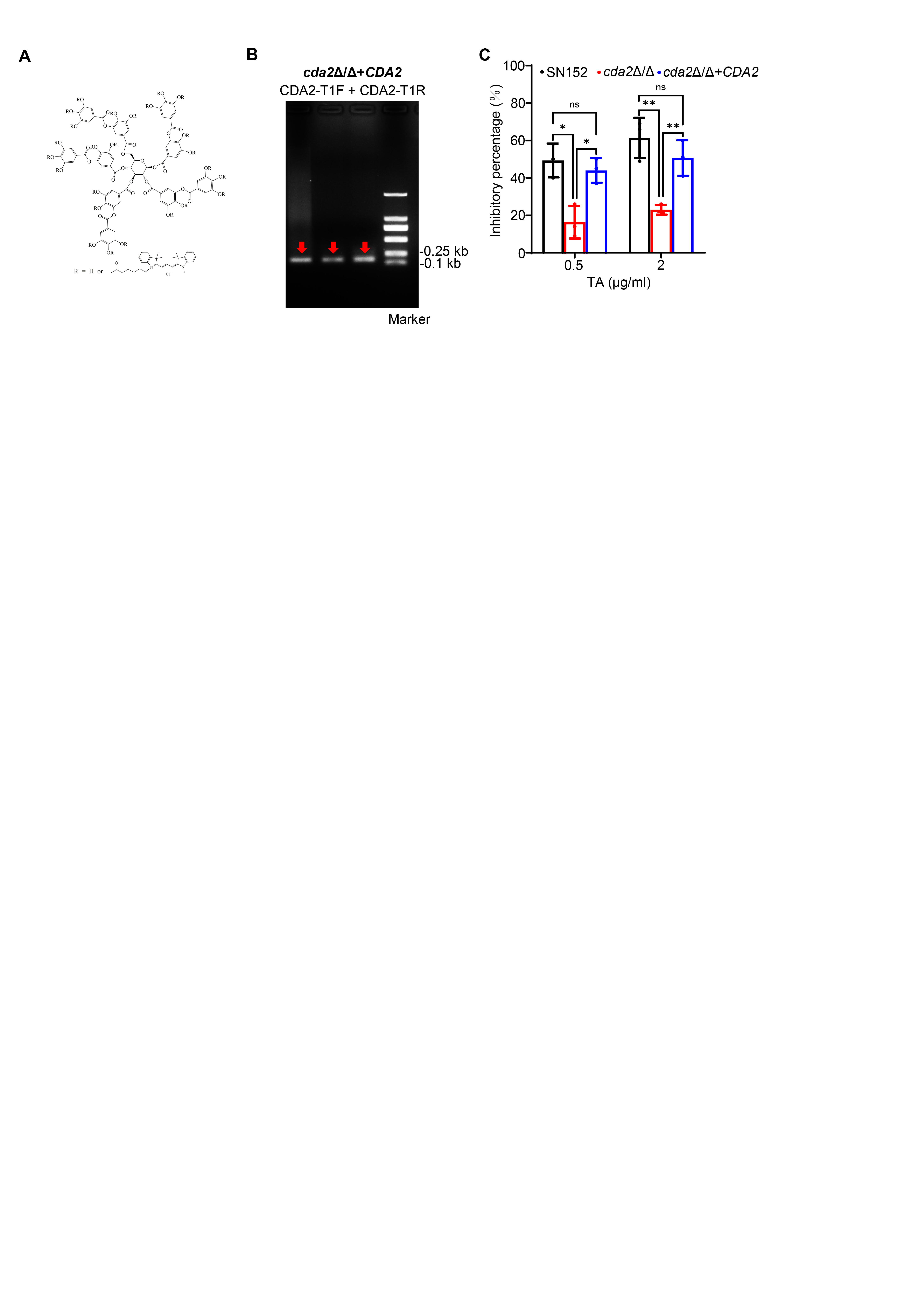

Supplement: S3 Fig — (A) Chemical structures of TA-Cy3. (B) Confirmation of the construction of CDA2 revertant mutant (cda2Δ/Δ + CDA2) identified by PCR. (C) Inhibitory percentage of TA against C. albicans SN152, cda2Δ/Δ and cda2Δ/Δ + CDA2. Data were shown as mean ± SD, two-tailed unpaired t test, *p < 0.05, **p < 0.01, ns, no significant. (TIF) [file ppat.1013596.s003.tif]

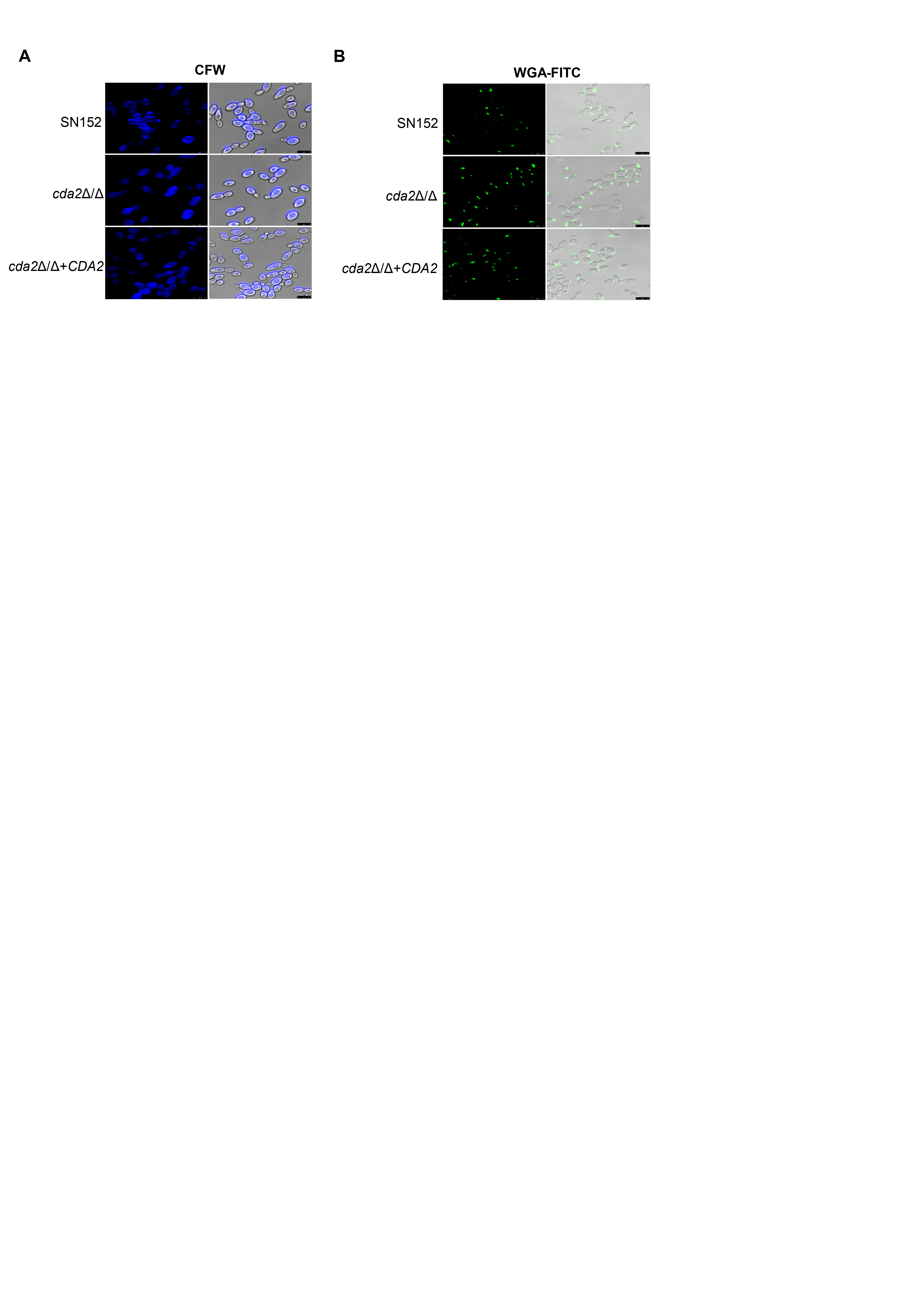

Supplement: S4 Fig — (A) Calcofluor white staining. (B)Wheat germ agglutinin staining. Yeast cells of C. albicans SN152, cda2Δ/Δ and cda2Δ/Δ + CDA2 were cultured in YPD at 30 °C for 6 h. Scale bars,7.5 μm. (TIF) [file ppat.1013596.s004.tif]
