# Supplementary material for: Dietary tannic acid promotes intestinal clearance of C. albicans by cross-linking hyphal chitosan
Source: PLoS Pathog. 2025 Oct 15;21(10):e1013596. doi: 10.1371/journal.ppat.1013596 (PMC12543286; doi:10.1371/journal.ppat.1013596)
Supplement: S1 Table — (DOCX) [file ppat.1013596.s005.docx]

Key sources table.

| REAGENT or RESOURCE | SOURCE | IDENTIFIER |
| --- | --- | --- |
| Strains and plasmids | | |
| *C. albicans* SC5314 | Lab stock | N/A |
| *C. albicans* SN152 | Lab stock | *arg4*Δ/ *arg4*Δ, *leu2*Δ/ *leu2*Δ, *his1*Δ/ *his1*Δ |
| *C. albicans cda2*Δ/Δ | This study | *cda2*Δ:: *HIS1/cda2*Δ:: *HIS1,arg4*Δ*/ arg4*Δ*, leu2*Δ*/ leu2*Δ*,* |
| *C. albicans cda2*Δ/Δ+*CAD2* | This study | *arg4*Δ:: *CIP10*-*ARG4*-*CDA2*/ *arg4*Δ:: *CIP10*-*ARG4*-*CDA2* *cda2*Δ:: *HIS1/ cda2*Δ:: *HIS1, leu2*Δ*/ leu2*Δ |
| *C. albicans tup1*Δ/Δ | ^1^ | *ura3*Δ::*imm434/ura3*Δ:: *imm434*, *tup1*Δ::*hisG*/*tup1*Δ::*hisG* |
| *C. albicans* *nrg1*Δ/Δ | ^2^ | *ura3*Δ::*imm434/ura3*Δ:: *imm434*, *nrg1*Δ::*hisG*/*nrg1*Δ::*hisG* |
| *C. albicans efg1*Δ/Δ*cph1*Δ/Δ | ^3^ | *ura3*Δ::*imm434*/*ura3*Δ:: *imm434*, *cph1*Δ::*hisG*/*cph1*Δ::*hisG* ,*efg1*Δ::*hisG*/*efg1*Δ::*hisG* |
| *C. albicans* 384 | Lab stock | N/A |
| *C. albicans* 388 | Lab stock | N/A |
| *C. albicans* 901 | Lab stock | N/A |
| *C. albicans* 904 | Lab stock | N/A |
| *C. albicans* 938 | Lab stock | N/A |
| *C. albicans* 939 | Lab stock | N/A |
| pV1093 | ^4^ | N/A |
| pFA-HA-HIS1 | Lab stock | N/A |
| pFA-HA-ARG4 | Lab stock | N/A |
| Trans5a Chemically Competent Cell | TransGen Biotech | Cat# CD201-02 |
| CIP10 | ^5^ | N/A |
| CIP10-ARG4 | This study | N/A |
| CIP10-ARG4-CDA2 | This study | N/A |
| Chemicals and enzymes | | |
| Yeast extract | Gibco | Cat# 212750 |
| Peptone | Gibco | Cat# 211693 |
| Glucose | Sangon Biotech | Cat# A501991-0500 |
| Agar | BD | Cat# 214010 |
| Yeast nitrogen base without amino acids | BD | Cat# DF0919-15-3 |
| Amino acids | Sigma | N/A |
| RPMI 1640 medium powder | Gibco | Cat# 31800022 |
| NaHCO_3_ | Sinopharm | Cat# 10018960 |
| NaOH | Greagent | Cat# G19852H |
| MOPS | Yuanye | Cat# S16036 |
| Nutrient broth | BD | Cat# 234000 |
| Tryptone | Thermo Scientific | Cat# LP0042B |
| NaCl | Greagent | Cat# G81793J |
| KOH | Sigma | Cat# 221473 |
| FITC -Dextran | Meilunbio | Cat# MB12674 |
| Amphotericin B | Meilunbio | Cat# MB1013 |
| KCl | Sinopharm | Cat# 10016318 |
| CaCl_2_ | Sinopharm | Cat# 10005861 |
| Congo red | Sigma-Aldrich | Cat# C6767 |
| Calcofluor white | Sigma-Aldrich | Cat# 18909 |
| Caspofungin | Meilunbio | Cat# MB2078 |
| Micafungin | Meilunbio | Cat# MB5545 |
| H_2_O_2_ | Sigma-Aldrich | Cat# H1009 |
| Fluconazole | Meilunbio | Cat# MB1288 |
| Miconazole | Sigma-Aldrich | Cat# 1443500 |
| Wheat germ agglutinin | Sigma-Aldrich | Cat# L4895 |
| DMEM | Gibco | Cat# 11320033 |
| Penicillin-streptomycin | Sigma-Aldrich | Cat# G6784 |
| FBS | Gibco | Cat# 26170043 |
| Propidium iodide | Sigma-Aldrich | Cat# P4170 |
| Levofloxacin hydrochloride | Yuanye | Cat# S26142 |
| Cyclophosphamide | Yuanye | Cat# S30563 |
| Tannic acid | TargetMol | Cat# T0801 |
| Naringin | Wokai | Cat# D16587 |
| Secoisolariciresinol diglucoside | Yuanye | Cat# B21419 |
| Secoisolariciresinol | Yuanye | Cat# B20666 |
| Resveratrol | Wokai | Cat# D11186 |
| Naringenin | Wokai | Cat# A28384 |
| Genistein | Yuanye | Cat# S31565 |
| Protocatechuic acid | Nature Standard | Cat# RS04101020 |
| Isoquercitrin | CATO | Cat# CCPE900102 |
| Caffeic acid | Wokai | Cat# D16602 |
| Rutin | Acros | Cat# C13239 |
| Gallic acid | Sinopharm | Cat# 10010218 |
| Catechin | Yuanye | Cat# B21722 |
| Ferulic acid | Yuanye | Cat# S31399 |
| Vanillic acid | Yuanye | Cat# S31038 |
| Hesperetin | Yuanye | Cat# S31386 |
| Kaempferol | Yuanye | Cat# S25632 |
| Diosmetin | Yuanye | Cat# S31450 |
| Quercetin | Yuanye | Cat# S25567 |
| Myricetin | Yuanye | Cat# S25978 |
| Chlorogenic acid | Yuanye | Cat# S30617 |
| Luteolin | Yuanye | Cat# S31366 |
| Apigenin | Yuanye | Cat# S31423 |
| Ellagic acid | Yuanye | Cat# S31544 |
| Ampicillin | Yuanye | Cat# A100339-0005 |
| Gentamicin | Yuanye | Cat# S17024 |
| DMSO | Sinopharm | Cat# 30072418 |
| Ethanol absolute | Sinopharm | Cat# 10009218 |
| Agarose | Biogreen | Cat# MB2788-1 |
| Goldview | Yuanye | Cat# R20977 |
| 50×TAE Buffer | Sangon Biotech | Cat# B548101-0500 |
| Yeastmaker Carrier DNA | Clontech | Cat# 630440 |
| 20×PBS | Meilunbio | Cat# MA0020 |
| CCK-8 | TargetMol | Cat# C0005 |
| Proteinase K Solution | Meilunbio | Cat# MA0006-1 |
| Chitosan oligosaccharide | Yuanye | Cat# S31060 |
| Eosin Y | Sangon Biotech | Cat# A600441-0025 |
| Na_2_HPO_4_ | Sangon Biotech | Cat# A610404-0100 |
| Citric acid | Sigma | Cat# 791725 |
| 4% Paraformaldehyde Fix Solution | Servicebio | Cat# G1101 |
| SpeedyCut SacI | Sangon Biotech | Cat# B600756-0100 |
| SpeedyCut XbaI | Sangon Biotech | Cat# B600766-0500 |
| SpeedyCut KpnI | Sangon Biotech | Cat# B600744-0200 |
| SpeedyCut StuI | Sangon Biotech | Cat# B600764-0100 |
| Premix Taq (Ex Taq Version 2.0) | Takara | Cat# RR003Q |
| CY3-COOH | Duofluor | Cat# D10122-25 |
| DMF | Sinopharm | Cat# 81007718 |
| DIC | Acros | Cat# C44618 |
| HOBt | Wokai | Cat# D10138 |
| DMAP | Sinopharm | Cat# 30198415 |
| Acetone | Sinopharm | Cat# 10000418 |
| Critical Commercial Assays | | |
| SanPrep Column DNA Gel Extraction Kit | Sangon Biotech | Cat# B518131-0100 |
| Rapid Yeast Genomic DNA Isolation Kit | Sangon Biotech | Cat# B518227-0100 |
| SanPrep Column PCR Product Purification Kit | Sangon Biotech | Cat# B518141-0100 |
| SanPrep Column Plasmid Mini-Preps Kit | Sangon Biotech | Cat# B518191-0100 |
| Yeastmaker Yeast Transformation System 2 | Clontech | Cat# 630439 |
| Fungal RNA Column extraction kit | Kanglang | Cat# KL-10112BTN |
| pEASY-Basic Seamless Cloning and Assembly Kit | TransGen Biotech | Cat# CU201 |
| Chitosan Assay Kit (Colorimetric) | Abcam | Cat# AC12559449 |
| Experimental Models: Organisms | | |
| Mouse: SPF mice (ICR, female) | Shanghai Regan Biotechnology Co., Ltd | N/A |
| Software and algorithms | | |
| GraphPad Prism 9.0 | GraphPad | https://www.graphpad.com/ |
| 3DHISTECH's Slide Converter | 3DHISTECH Ltd. | https://www.3dhistech.com/research/slidecenter/slidemaster/ |
| Tanon 1600 Gel Imaging System | Tanon | N/A |
| Leica LAS AF Lite 2.6.1 | Leica | N/A |
| Origin2021 | OriginLab | https://www.originlab.com/ |
| Ascent Software Version 2.6 | Thermo Fisher Scientific | N/A |
| DESeq2 Version 1.40.0 | Bioconductor | https://bioconductor.org/packages/release/bioc/html/DESeq2.html |
| SnapGene (version 4.3.6) | SnapGene | https://www.snapgene.com/ |

**Table S1. Primers used in this study.**

| **Name** | **Sequence** | **Discription** |
| --- | --- | --- |
| P7 | ATCTCATTAGATTTGGAACTTGTGGGTT | *CAS9* gene |
| P8 | TTCGAGCGTCCCAAAACCTTCT | *CAS9* gene |
| P1 | AAGAAAGAAAGAAAACCAGGAGTGAA | sgRNA |
| P4 | ACAAATATTTAAACTCGGGACCTGG | sgRNA |
| P5 | GCGGCCGCAAGTGATTAGACT | sgRNA |
| P6 | GCAGCTCAGTGATTAAGAGTAAAGATGG | sgRNA |
| CDA2-sg-F | ACTTTACTGGATTACATGAAGTTTTAGAGCTAGAAATAGCAAGTTAAA | sgRNA for *CDA2* |
| CDA2-sg-R | TTCATGTAATCCAGTAAAGTCAAATTAAAAATAGTTTACGCAAGTC | sgRNA for *CDA2* |
| CDA2-Re-F | AGATAGTAGTAGTTCTAGTGGTGGTGGTGGCGGCAGTGGATTACCACCTTCTCCAATGCATCCAATACCTCGCCAGAAC | repair DNA for *CDA2* |
| CDA2-Re-R | CATAAGGTGGTCGAAACCATTTTGGTAAATGATTCAGAGTAGCATTCATGGCCCAAATTGCTCACTATAGGGAGACCG | repair DNA for *CDA2* |
| CDA2-Te-F | GGTCCAGCCACAATAAATAG | genotype confirmation for *CDA2* |
| CDA2-Te-R | CACCACCACCACCAAGCATT | genotype confirmation for *CDA2* |
| CDA2-N1F | TCGACCTCGAGGGGGGGCCCGGTACCAATCACTTCAACTCCAACTACGA | amplification of *CDA2* gene |
| CDA2-N1R | TCGGCTTTACTTGCTTGGATTGCTACTAAAGGGAACAAAAGCTGGGTAC | amplification of *CDA2* gene |
| CDA2-T1F | GCAACAACAACAACAACCT | genotype confirmation for *CDA2* |
| CDA2-T1R | ATGTTGATGTTGATGGTCC | genotype confirmation for *CDA2* |
| ARG4-N1F | ACTCACTATAGGGCGAATTGGAGCTTTCACCTACGCTGTTCTTT | amplification of *ARG4* gene |
| ARG4-N1R | GAGGAGAAGAATCAGAACGCGATCTGGGGGATCCACTAGTTCTAG | amplification of *ARG4* gene |

**References**

1. Mao X, Li Y, Wang H, Cao F, Chen J. Antagonistic interplay of Swi1 and Tup1 on filamentous growth of Candida albicans. FEMS microbiology letters 2008; 285:233-41.

2. Braun BR, Kadosh D, Johnson AD. NRG1, a repressor of filamentous growth in C. albicans, is down‐regulated during filament induction. The EMBO journal 2001.

3. Lv Q-Z, Li D-D, Han H, Yang Y-H, Duan J-L, Ma H-H, et al. Priming with FLO8-deficient Candida albicans induces Th1-biased protective immunity against lethal polymicrobial sepsis. Cellular & Molecular Immunology 2021; 18:2010-23.

4. Vyas VK, Barrasa MI, Fink GR. A Candida albicans CRISPR system permits genetic engineering of essential genes and gene families. Science advances 2015; 1:e1500248.

5. Alam MR, Caldwell BD, Johnson RC, Darlington DN, Mains RE, Eipper BA. Novel proteins that interact with the COOH-terminal cytosolic routing determinants of an integral membrane peptide-processing enzyme. Journal of Biological Chemistry 1996; 271:28636-40.
