# Supplementary material for: Dietary tannic acid promotes intestinal clearance of C. albicans by cross-linking hyphal chitosan
Source: PLoS Pathog. 2025 Oct 15;21(10):e1013596. doi: 10.1371/journal.ppat.1013596 (PMC12543286; doi:10.1371/journal.ppat.1013596)
Supplement: S1 Data — Table containing the underlying data for Figs 1B, 1C, 1F, 1H, 1I, 2A, 2C, 2D–F, 2H–J, 3J, 4B, 4D–F, 4H, 4I, 6A, 6C, 6E, 6G, S1A, S1C, S1F and S3C. (DOCX) [file ppat.1013596.s006.docx]

| Fig 1B Minimum inhibitory concentrations (MICs) of 24 dietary polyphenols | | |
| --- | --- | --- |
| Chemical compound | **MIC (μg/ml)** | |
|  | ***C. albicans* SC5314** | ***C. albicans* 904** |
| Naringin | ＞128 | ＞128 |
| Resveratrol | ＞128 | ＞128 |
| Naringenin | ＞128 | ＞128 |
| Genistein | ＞128 | ＞128 |
| Protocatechuic acid | ＞128 | ＞128 |
| Isoquercitrin | ＞128 | ＞128 |
| Caffeic acid | ＞128 | ＞128 |
| Rutin | ＞128 | ＞128 |
| Gallic acid | ＞128 | ＞128 |
| Catechin | ＞128 | ＞128 |
| Ferulic acid | ＞128 | ＞128 |
| Vanillic acid | ＞128 | ＞128 |
| Hesperetin | ＞128 | ＞128 |
| Kaempferol | ＞128 | ＞128 |
| Diosmetin | ＞128 | ＞128 |
| Quercetin | ＞128 | ＞128 |
| Myricetin | 64 | 64 |
| Tannic acid | 4 | 4 |
| Chlorogenic acid | ＞128 | ＞128 |
| Luteolin | ＞128 | ＞128 |
| Apigenin | ＞128 | ＞128 |
| Ellagic acid | 32 | 32 |
| Secoisolariciresinol diglucoside | ＞128 | ＞128 |
| Secoisolariciresinol | ＞128 | ＞128 |

| Fig 1C Tannic acid (TA) against standard or clinical isolated *C. albicans* 384, 388, 901, 939. | |
| --- | --- |
| *C. albicans* | **MIC (μg/ml)** |
| 384 | 4 |
|  | 4 |
| 388 | 4 |
|  | 4 |
| 901 | 4 |
|  | 4 |
| 939 | 4 |
|  | 4 |

| Fig 1F Fungal burden of jejunum, ileum and colon (n=6) | | | |
| --- | --- | --- | --- |
| Organ | **Days post-infection** | **Fungal colony-forming units (CFUs) (×10^4^/g)** | |
|  |  | **Con** | **TA 20 mg/kg** |
| Jejunum | **1** | 5.093 | 0.123 |
|  |  | 4.092 | 0.316 |
|  |  | 1.093 | 1.044 |
|  |  | 9.961 | 1.327 |
|  |  | 2.028 | 1.032 |
|  |  | 10.026 | 0.179 |
|  | **2** | 1.838 | 4.198 |
|  |  | 3.499 | 5.781 |
|  |  | 5.721 | 2.128 |
|  |  | 3.436 | 8.047 |
|  |  | 2.715 | 1.274 |
|  |  | 17.009 | 2.052 |
|  | **4** | 15.054 | 2.879 |
|  |  | 30.39 | 26.985 |
|  |  | 37.823 | 22.011 |
|  |  | 20.006 | 41.119 |
|  |  | 30.401 | 36.547 |
|  |  | 25.476 | 26.811 |
| Ileum | **1** | 13.688 | 1.05 |
|  |  | 5.87 | 0.605 |
|  |  | 4.082 | 0.447 |
|  |  | 20.455 | 0.863 |
|  |  | 2.947 | 0.413 |
|  |  | 1.449 | 0.023 |
|  | **2** | 20.536 | 12.416 |
|  |  | 38.969 | 29.8 |
|  |  | 47.136 | 0.619 |
|  |  | 92.141 | 8.312 |
|  |  | 22.004 | 6.882 |
|  |  | 54.027 | 0.745 |
|  | **4** | 65.227 | 40.442 |
|  |  | 50.111 | 51.754 |
|  |  | 75.683 | 8.974 |
|  |  | 50.744 | 73.446 |
|  |  | 106.11 | 53.508 |
|  |  | 90.679 | 30.515 |
| Colon | **1** | 1.534 | 1.446 |
|  |  | 2.505 | 1.289 |
|  |  | 5.46 | 2.098 |
|  |  | 13.616 | 0.749 |
|  |  | 5.3447 | 2.301 |
|  |  | 6.142 | 1.104 |
|  | **2** | 1.132 | 0.339 |
|  |  | 1.334 | 8.002 |
|  |  | 12.852 | 1.16 |
|  |  | 3.006 | 8.322 |
|  |  | 1.435 | 1.086 |
|  |  | 21.1 | 16.271 |
|  | **4** | 20.837 | 20.937 |
|  |  | 30.446 | 64.281 |
|  |  | 43.387 | 5.476 |
|  |  | 5.566 | 10.176 |
|  |  | 44.896 | 32.949 |
|  |  | 13.66 | 25 |

| Fig 1H Plasma FITC-Dextran fluorescence intensity (n=5) | | |
| --- | --- | --- |
| FITC-Dextran fluorescence intensity (RLU) | | |
| Uninfected | ***C. a*** | ***C. a* +TA 20 mg/kg** |
| 328.5 | 702.5 | 552.5 |
| 535.5 | 1427.5 | 335.5 |
| 543.5 | 1211.5 | 511 |
| 726.5 | 554 | 377 |
| 429.5 | 1334 | 634 |

| Fig 1I Fecal fungal burden (n=6) | | |
| --- | --- | --- |
| Days post-infection | **CFU (×10^5^/g)** | |
|  | **Con** | **TA 20 mg/kg** |
| 1 | 20.174 | 92.399 |
|  | 23.606 | 67.19 |
|  | 48.22 | 75.398 |
|  | 69.922 | 94.871 |
|  | 62.703 | 84.904 |
|  | 63.477 | 38.497 |
| 2 | 0.027 | 1.015 |
|  | 0.217 | 0.476 |
|  | 0.288 | 0.582 |
|  | 0.121 | 1.221 |
|  | 0.034 | 2.845 |
|  | 0.053 | 0.218 |
| 4 | 0.189 | 0.26 |
|  | 0.098 | 0.241 |
|  | 0.289 | 0.837 |
|  | 0.041 | 0.905 |
|  | 0.395 | 0.259 |
|  | 0.609 | 0.388 |
| 6 | 0.544 | 6.39 |
|  | 0.421 | 1.849 |
|  | 0.758 | 1.519 |
|  | 1.049 | 1.24 |
|  | 2.176 | 2.545 |
|  | 3.014 | 1.62 |
| 8 | 30.035 | 1.2 |
|  | 15.178 | 2.52 |
|  | 91.27 | 4.607 |
|  | 92.42 | 8.373 |
|  | 82.29 | 1.436 |
|  | 66.83 | 3.62 |

| Fig 2A Time-growth curves in *C. albicans* SC5314 (YPD, 37 ℃) | | | |
| --- | --- | --- | --- |
| Time (h) | **OD_600_** | | |
|  | **Con** | **TA 16 μg/ml** | **TA 64 μg/ml** |
| 0 | 0.1 | 0.1 | 0.1 |
|  | 0.1 | 0.1 | 0.1 |
|  | 0.1 | 0.1 | 0.1 |
| 2 | 0.22 | 0.28 | 0.35 |
|  | 0.2 | 0.24 | 0.3 |
|  | 0.22 | 0.24 | 0.3 |
| 4 | 0.54 | 0.59 | 0.6 |
|  | 0.5 | 0.54 | 0.6 |
|  | 0.51 | 0.54 | 0.68 |
| 6 | 2.32 | 2.28 | 2.12 |
|  | 2.08 | 2.04 | 1.96 |
|  | 2.16 | 2.08 | 1.88 |
| 8 | 4.02 | 3.78 | 3.36 |
|  | 3.84 | 3.66 | 3.06 |
|  | 3.72 | 3.6 | 3.12 |
| 10 | 6.7 | 6.7 | 5.8 |
|  | 6.9 | 6.8 | 5.6 |
|  | 6.6 | 6.7 | 5.6 |
| 12 | 9.4 | 9.6 | 8.2 |
|  | 9.6 | 8.6 | 8.4 |
|  | 9.6 | 8.8 | 8.8 |
| 24 | 10.2 | 11.4 | 9.2 |
|  | 9.8 | 9.6 | 9 |
|  | 9.6 | 9.6 | 9 |

| Fig 2C The wet weight ratio of *C. albicans* treated with TA and ddH_2_O (RPMI 1640, 37 ℃) | | | |
| --- | --- | --- | --- |
| Time (h) | **Treatment/Control (w/w, %)** | | |
|  | **TA 0.125 μg/ml** | **TA 0.25 μg/ml** | **TA 0.5 μg/ml** |
| 3 | 84 | 93 | 87 |
|  | 86 | 66 | 81 |
|  | 85 | 73 | 96 |
| 6 | 90 | 79 | 94 |
|  | 97 | 78 | 97 |
|  | 90 | 82 | 81 |
| 9 | 86 | 75 | 85 |
|  | 89 | 85 | 57 |
|  | 84 | 71 | 58 |
| 12 | 85 | 74 | 44 |
|  | 72 | 72 | 63 |
|  | 96 | 70 | 58 |
| 24 | 67 | 44 | 29 |
|  | 90 | 64 | 37 |
|  | 72 | 68 | 41 |

| Fig 2D Wet weights of clinical isolated *C. albicans* 388, 901, 938, 939 in the presence of TA, 24 h) | | | | |
| --- | --- | --- | --- | --- |
| *C. albicans* | **Wet weight (mg)** | | | |
|  | **Con** | **TA 0.125 μg/ml** | **TA 0.25 μg/ml** | **TA 0.5 μg/ml** |
| 388 | 22.8 | 12.8 | 12.5 | 11.2 |
|  | 26.8 | 12.8 | 9.6 | 7 |
|  | 24.8 | 12.8 | 10.9 | 10.8 |
| 901 | 15.2 | 14.9 | 8.4 | 7.8 |
|  | 14.7 | 13.2 | 11.6 | 10 |
|  | 13.7 | 13.5 | 10.6 | 9.7 |
| 938 | 37.2 | 29.2 | 25.2 | 17.5 |
|  | 35.4 | 30.2 | 29.8 | 14.9 |
|  | 37.2 | 27.2 | 25.1 | 21.8 |
| 939 | 38.9 | 38.9 | 26.9 | 25.4 |
|  | 40.8 | 33.4 | 28.8 | 20.9 |
|  | 35.1 | 30.6 | 28.6 | 27.7 |

| Fig 2F Wet weights of hyphae-blocked *tup1*Δ/Δ and *nrg1*Δ/Δ mutants (YPD, 37 ℃) | | | | |
| --- | --- | --- | --- | --- |
| *C. albicans* | **Wet weight (mg)** | | | |
|  | **Con** | **TA 0.125 μg/ml** | **TA 0.25 μg/ml** | **TA 0.5 μg/ml** |
| *tup1*Δ/Δ | 9 | 6.4 | 4.3 | 3.6 |
|  | 14.4 | 5.2 | 4.8 | 3 |
|  | 12 | 6.3 | 3.3 | 2.7 |
| *nrg1*Δ/Δ | 116.5 | 68.9 | 52.7 | 48 |
|  | 121.9 | 58.3 | 57.4 | 48.7 |
|  | 114.3 | 63.4 | 59.6 | 54.3 |

| Fig 2E The proliferation of yeast-blocked *efg1*Δ/Δ*cph1*Δ/Δ mutant (RPMI 1640, 37 ℃) | | | |
| --- | --- | --- | --- |
| Time (h) | **OD_600_** | | |
|  | **Con** | **TA 16 μg/ml** | **TA 64 μg/ml** |
| 0 | 0.1 | 0.1 | 0.1 |
|  | 0.1 | 0.1 | 0.1 |
|  | 0.1 | 0.1 | 0.1 |
| 2 | 0.23 | 0.24 | 0.26 |
|  | 0.21 | 0.24 | 0.24 |
|  | 0.28 | 0.28 | 0.29 |
| 4 | 0.5 | 0.49 | 0.51 |
|  | 0.45 | 0.44 | 0.45 |
|  | 0.54 | 0.53 | 0.53 |
| 6 | 1.12 | 1.06 | 1.08 |
|  | 1.02 | 0.98 | 0.96 |
|  | 1.24 | 1.16 | 1.18 |
| 8 | 1.44 | 1.32 | 1.36 |
|  | 1.4 | 1.4 | 1.36 |
|  | 1.72 | 1.64 | 1.72 |
| 10 | 2 | 1.8 | 2 |
|  | 2 | 1.7 | 1.7 |
|  | 2.3 | 2.1 | 2 |
| 12 | 2.2 | 1.9 | 2.2 |
|  | 2.2 | 1.9 | 2 |
|  | 2.4 | 2.3 | 2.3 |
| 24 | 2.4 | 2.2 | 2.3 |
|  | 2.2 | 2.4 | 2.4 |
|  | 2.3 | 2 | 2.1 |

| Fig 2H Wet weights of shaking or static *tup1*Δ/Δ and *nrg1*Δ/Δ mutants treated with TA (RPMI 1640, 37 ℃) | | | | | |
| --- | --- | --- | --- | --- | --- |
| *C. albicans* | **Time (h)** | **Wet weight (mg)** | | | |
|  |  | **Con** | **TA 0.125 μg/ml** | **TA 0.25 μg/ml** | **TA 0.5 μg/ml** |
| *tup1*Δ/Δ shaking | **12** | 5.7 | 1.7 | 0.2 | 0.1 |
|  |  | 3.5 | 1 | 0.3 | 0.2 |
|  |  | 4.8 | 1 | 0.3 | 0.1 |
|  | **24** | 8.5 | 1.8 | 0.6 | 0.2 |
|  |  | 9.3 | 1.2 | 0.7 | 0.2 |
|  |  | 8.1 | 1.5 | 1 | 0.3 |
| *nrg1*Δ/Δ shaking | **12** | 5.8 | 2 | 1.1 | 0.1 |
|  |  | 7.8 | 2.5 | 2 | 0.4 |
|  |  | 5.5 | 1.5 | 1 | 0.3 |
|  | **24** | 17.3 | 5.8 | 5.2 | 3.7 |
|  |  | 16.5 | 6 | 4.5 | 4 |
|  |  | 18.4 | 4.4 | 4 | 3.5 |

| Fig 2H Wet weights of shaking or static *tup1*Δ/Δ and *nrg1*Δ/Δ mutants treated with TA (RPMI 1640, 37 ℃) | | | | | |
| --- | --- | --- | --- | --- | --- |
| *C. albicans* | **Time (h)** | **Wet weight (mg)** | | | |
|  |  | **Con** | **TA 0.25 μg/ml** | **TA 0.5 μg/ml** | **TA 1 μg/ml** |
| *tup1*Δ/Δ static | **24** | 10 | 9 | 8.2 | 7.5 |
|  |  | 12.8 | 10.8 | 5.6 | 4.3 |
|  |  | 11.6 | 7.9 | 5.9 | 6.8 |
|  | **48** | 39.5 | 26.5 | 16.4 | 5 |
|  |  | 20.5 | 17.7 | 15.4 | 5.3 |
|  |  | 23.3 | 17.6 | 14.8 | 4.5 |

| Fig 2H Wet weights of shaking or static *tup1*Δ/Δ and *nrg1*Δ/Δ mutants treated with TA (RPMI 1640, 37 ℃) | | | | | |
| --- | --- | --- | --- | --- | --- |
| *C. albicans* | **Time (h)** | **Wet weight (mg)** | | | |
|  |  | **Con** | **TA 1 μg/ml** | **TA 4 μg/ml** | **TA 16 μg/ml** |
| *nrg1*Δ/Δ static | **24** | 57.8 | 42.5 | 35.3 | 35.8 |
|  |  | 58.1 | 49.9 | 49.6 | 36 |
|  |  | 54.6 | 51.9 | 43.1 | 27.7 |
|  | **48** | 98.7 | 58.2 | 48.6 | 47.6 |
|  |  | 104.4 | 65.2 | 64 | 52.1 |
|  |  | 80.9 | 65.2 | 64.5 | 51.9 |

| Fig 2I Metabolic activities assayed by CCK-8 | | | |
| --- | --- | --- | --- |
| OD_450_ | | | |
| Con | **TA 0.125 μg/ml** | **TA 0.25 μg/ml** | **TA 0.5 μg/ml** |
|  |  |  |  |
|  |  |  |  |
|  |  |  |  |

| Fig 2I Metabolic activities assayed by CCK-8 | | | |
| --- | --- | --- | --- |
| OD_450_ | | | |
| Con | **TA 0.125 μg/ml** | **TA 0.25 μg/ml** | **TA 0.5 μg/ml** |
|  |  |  |  |
|  |  |  |  |
|  |  |  |  |

| Fig 2I Metabolic activities assayed by CCK-8 | | | |
| --- | --- | --- | --- |
| OD_450_ | | | |
| Con | **TA 0.125 μg/ml** | **TA 0.25 μg/ml** | **TA 0.5 μg/ml** |
| 3.08 | 3.147 | 2.94 | 2.955 |
| 3.198 | 3.172 | 3.172 | 3.147 |
| 2.983 | 3.175 | 2.939 | 3.015 |

| Fig 2J RAW 264.7 cells were co-incubated with *C. albicans* SC5314 | | | |
| --- | --- | --- | --- |
| % of P^+^ cells | | | |
| Con | **TA 0.5 μg/ml** | **TA 2 μg/ml** | **TA 8 μg/ml** |
| 65.2 | 9.4 | 8.2 | 7 |
| 48.3 | 16.7 | 7.5 | 5.8 |
| 41.1 | 10.6 | 11.8 | 9 |

| Fig 3J Inhibitory percentages of TA against parental SN152 and *chs3*Δ/Δ, *cda2*Δ/Δ mutants | | |
| --- | --- | --- |
| *C. albicans* | **Inhibitory percentage (%)** | |
|  | **TA 0.5 μg/ml** | **TA 2 μg/ml** |
| SN152 | 66 | 66 |
|  | 60 | 60 |
|  | 54 | 55 |
|  | 69 | 62 |
|  | 66 | 65 |
|  | 59 | 68 |
| *cda2*Δ/Δ | 27 | 17 |
|  | 23 | 27 |
|  | 25 | 29 |
|  | 28 | 27 |
|  | 24 | 26 |
|  | 16 | 10 |

| Fig 4B ELISA quantification of chitosan in cell wall extracts from *C. albicans* SN152, *cda2*Δ/Δ and *cda2*Δ/Δ+*CDA2* | | |
| --- | --- | --- |
| *C. albicans* | **Chitosan (μg/g)** | |
|  | **Yeast** | **Hyphae** |
| SN152 | 91.585 | 159.34 |
|  | 89.151 | 129.925 |
|  | 85.214 | 139.372 |
| *cda2*Δ/Δ | 71.543 | 43.741 |
|  | 78.072 | 38.695 |
|  | 79.448 | 22.753 |
| *cda2*Δ/Δ+*CDA2* | 95.486 | 165.449 |
|  | 82.202 | 135.776 |
|  | 83.323 | 120.432 |

| Fig 4E Fungal burden of ileum and colon (n=6) | | | |
| --- | --- | --- | --- |
| Organ | ***C. albicans*** | **CFU (×10^3^/g)** | |
|  |  | **Con** | **TA 20 mg/kg** |
| Ileum | **SN152** | 3.251 | 5.43 |
|  |  | 16.05 | 2.515 |
|  |  | 12.053 | 0.685 |
|  |  | 7.149 | 0.532 |
|  |  | 9.247 | 0.479 |
|  |  | 5.23 | 2.84 |
|  | ***cda2*Δ/Δ** | 1.965 | 0.531 |
|  |  | 0.517 | 1.034 |
|  |  | 3.689 | 3.652 |
|  |  | 0.67 | 0.675 |
|  |  | 2.179 | 2.132 |
|  |  | 0.593 | 0.812 |
|  | ***cda2*Δ/Δ+*CDA2*** | 9.74 | 0.31 |
|  |  | 5.908 | 0.864 |
|  |  | 4.608 | 1.319 |
|  |  | 10.446 | 0.874 |
|  |  | 7.193 | 5.181 |
|  |  | 11.719 | 7.137 |
| Colon | **SN152** | 0.902 | 0.208 |
|  |  | 1.437 | 1.754 |
|  |  | 1.358 | 1.034 |
|  |  | 1.772 | 3.116 |
|  |  | 2.776 | 2.06 |
|  |  | 2.897 | 1.357 |
|  | ***cda2*Δ/Δ** | 3.711 | 0.609 |
|  |  | 1.587 | 1.047 |
|  |  | 1.173 | 2.155 |
|  |  | 1.206 | 2.367 |
|  |  | 0.823 | 1.581 |
|  |  | 1.139 | 1.136 |
|  | ***cda2*Δ/Δ+*CDA2*** | 0.961 | 1.116 |
|  |  | 2.547 | 2.043 |
|  |  | 1.072 | 2.157 |
|  |  | 1.118 | 1.325 |
|  |  | 1.868 | 0.56 |
|  |  | 2.688 | 3.017 |

| Fig 4F Fungal burden of feces (n=6) | | |
| --- | --- | --- |
| *C. albicans* | **CFU (×10^4^/g)** | |
|  | **Con** | **TA 20 mg/kg** |
| SN152 | 6.109 | 6.503 |
|  | 3.442 | 2.179 |
|  | 2.585 | 2.994 |
|  | 6.124 | 7.913 |
|  | 2.399 | 12.794 |
|  | 4.778 | 10.46 |
| *cda2*Δ/Δ | 5.911 | 3.258 |
|  | 8.207 | 6.451 |
|  | 9.618 | 3.347 |
|  | 7.145 | 5.28 |
|  | 13.098 | 12.643 |
|  | 6.255 | 10.332 |
| *cda2*Δ/Δ+*CDA2* | 4.736 | 10.476 |
|  | 2.737 | 3.591 |
|  | 1.531 | 4.679 |
|  | 3.145 | 9.215 |
|  | 5.17 | 6 |
|  | 1.135 | 12.319 |

| Fig 4H Time-growth curves of *C. albicans* SN152, *cda2*Δ/Δ and *cda2*Δ/Δ+*CDA2* cultured in YPD media at 30 ℃ | | | |
| --- | --- | --- | --- |
| Time (h) | **OD_600_** | | |
|  | **SN152** | ***cda2*Δ/Δ** | ***cda2*Δ/Δ+*CDA2*** |
| 0 | 0.1 | 0.1 | 0.1 |
|  | 0.1 | 0.1 | 0.1 |
|  | 0.1 | 0.1 | 0.1 |
| 3 | 0.23 | 0.11 | 0.22 |
|  | 0.23 | 0.13 | 0.22 |
|  | 0.25 | 0.12 | 0.22 |
| 6 | 1 | 0.6 | 0.92 |
|  | 0.96 | 0.6 | 0.9 |
|  | 0.92 | 0.52 | 0.92 |
| 9 | 2.7 | 1.4 | 2.5 |
|  | 2.5 | 1.5 | 2.3 |
|  | 2.9 | 1.4 | 2.2 |
| 12 | 7.8 | 4 | 7 |
|  | 7.2 | 3.8 | 6.4 |
|  | 6.8 | 2 | 6 |

| Fig 4I Wet weights of *C. albicans* SN152, *cda2*Δ/Δ and *cda2*Δ/Δ+*CDA2* cultured in RPMI 1640 at 37 ℃ | | | |
| --- | --- | --- | --- |
| Time (h) | **Wet weight (mg)** | | |
|  | **SN152** | ***cda2*Δ/Δ** | ***cda2*Δ/Δ+*CDA2*** |
| 12 | 30.4 | 20 | 31.5 |
|  | 35 | 18.4 | 37.2 |
|  | 37.3 | 16.9 | 31.1 |
| 24 | 41 | 22 | 38.7 |
|  | 42.5 | 17.7 | 44.1 |
|  | 36.8 | 30.4 | 43.8 |

| Fig 6A Hyphal germination and elongation | | | |
| --- | --- | --- | --- |
| Time (h) | **Hyphal length (μm)** | | |
|  | **SN152** | ***cda2*Δ/Δ** | ***cda2*Δ/Δ+*CDA2*** |
| 2 | 20.858 | 5.812 | 22.49 |
|  | 14.108 | 0 | 24.338 |
|  | 11.546 | 0 | 10.548 |
|  | 13.499 | 0 | 13.193 |
|  | 12.292 | 0 | 15.494 |
|  | 21.558 | 5.485 | 22.654 |
|  | 8.466 | 0 | 11.403 |
|  | 6.146 | 0 | 25.577 |
|  | 11.869 | 0 | 21.679 |
|  | 10.413 | 0 | 12.23 |
|  | 16.216 | 0 | 25.903 |
|  | 20.814 | 0 | 19.774 |
|  | 9.707 | 0 | 10.394 |
|  | 19.436 | 0 | 11.866 |
|  | 14.173 | 3.663 | 11.63 |
|  | 21.334 | 0 | 17.66 |
|  | 14.88 | 0 | 7.514 |
|  | 12.292 | 0 | 13.589 |
|  | 8.055 | 0 | 15.494 |
|  | 21.837 | 4.81 | 16.364 |
|  | 22.46 | 0 | 12.506 |
|  | 14.253 | 4.712 | 8.843 |
|  | 20.099 | 2.886 | 20.698 |
|  | 24.476 | 4.906 | 9.657 |
|  | 6.667 | 0 | 23.868 |
|  | 20.089 | 1.924 | 12.781 |
|  | 5.405 | 5.313 | 6.7 |
|  | 21.592 | 4.356 | 22.79 |
|  | 20.407 | 3.848 | 6.929 |
|  | 12.165 | 0 | 9.065 |
|  | 21.366 | 6.084 | 18.95 |
|  | 13.275 | 4.952 | 17.223 |
|  | 19.102 | 7.004 | 18.546 |
|  | 21.27 | 0 | 20.076 |
|  | 11.869 | 3.042 | 12.155 |
|  | 19.952 | 6.453 | 9.811 |
|  | 10.999 | 4.302 | 6.418 |
|  | 21.081 | 0 | 20.064 |
|  | 15.202 | 0 | 24.281 |
|  | 9.707 | 0 | 15.524 |
| 3 | 34.187 | 22.816 | 32.765 |
|  | 15.541 | 31.024 | 32.97 |
|  | 37.471 | 13.487 | 37.518 |
|  | 13.4 | 14.333 | 18.295 |
|  | 31.558 | 8.163 | 38.15 |
|  | 30.578 | 23.139 | 28.048 |
|  | 10.832 | 32.709 | 24.051 |
|  | 19.048 | 20.843 | 20.931 |
|  | 13.605 | 12.249 | 34.653 |
|  | 23.965 | 4.365 | 40.179 |
|  | 39.102 | 14.606 | 27.99 |
|  | 12.459 | 17.739 | 7.514 |
|  | 14.431 | 12.529 | 22.627 |
|  | 23.575 | 23.082 | 12.249 |
|  | 30.612 | 26.842 | 13.326 |
|  | 27.736 | 6.937 | 14.919 |
|  | 30.368 | 6.418 | 10.969 |
|  | 17.007 | 8.154 | 13.487 |
|  | 7.606 | 12.492 | 8.701 |
|  | 30.386 | 21.099 | 14.88 |
|  | 14.88 | 17.804 | 8.276 |
|  | 23.426 | 4.905 | 11.074 |
|  | 27.456 | 17.99 | 34.832 |
|  | 13.554 | 15.527 | 10.635 |
|  | 31.439 | 16.897 | 9.19 |
|  | 16.619 | 9.74 | 28.31 |
|  | 32.908 | 33.176 | 37.734 |
|  | 14.654 | 19.61 | 16.877 |
|  | 35.4 | 21.186 | 6.446 |
|  | 37.617 | 10.756 | 10.292 |
|  | 15.599 | 4.082 | 29.566 |
|  | 33.383 | 15.086 | 22.705 |
|  | 12.544 | 14.007 | 16.713 |
|  | 11.545 | 11.403 | 21.486 |
|  | 22.515 | 9.127 | 26.003 |
|  | 9.19 | 5.582 | 44.923 |
|  | 28.386 | 13.758 | 18.913 |
|  | 28.604 | 5.478 | 11.571 |
|  | 26.539 | 5.58 | 23.241 |
|  | 18.657 | 16.453 | 36.94 |
| 4 | 57.016 | 32.199 | 59.168 |
|  | 57.352 | 24.293 | 36.712 |
|  | 23.895 | 30.338 | 11.399 |
|  | 36.884 | 18.276 | 51.347 |
|  | 14.4 | 15.648 | 30.761 |
|  | 46.957 | 19.721 | 48.103 |
|  | 37.627 | 10.721 | 48.706 |
|  | 7.326 | 13.69 | 14.529 |
|  | 11.399 | 20.729 | 35.616 |
|  | 38.288 | 16.495 | 23.014 |
|  | 55.118 | 20.559 | 12.781 |
|  | 19.203 | 15.528 | 18.632 |
|  | 39.435 | 8.304 | 56.167 |
|  | 34.005 | 45.848 | 47.71 |
|  | 17.502 | 11.337 | 16.944 |
|  | 12.054 | 36.859 | 41.093 |
|  | 12.834 | 28.62 | 44.271 |
|  | 47.76 | 15.104 | 42.581 |
|  | 36.82 | 26.036 | 25.147 |
|  | 30.136 | 22.056 | 48.649 |
|  | 43.584 | 7.809 | 47.797 |
|  | 30.572 | 39.065 | 14.448 |
|  | 23.127 | 27.091 | 51.879 |
|  | 53.498 | 14.785 | 54.537 |
|  | 53.533 | 24.903 | 28.612 |
|  | 39.252 | 17.926 | 27.965 |
|  | 47.789 | 24.64 | 45.787 |
|  | 42.832 | 17.7 | 59.047 |
|  | 33.693 | 37.414 | 43.172 |
|  | 56.996 | 27.813 | 18.695 |
|  | 21.777 | 14.786 | 17.006 |
|  | 28.218 | 16.17 | 31.237 |
|  | 20.508 | 16.338 | 40.225 |
|  | 35.956 | 17.542 | 18.135 |
|  | 30.63 | 23.972 | 39.637 |
|  | 13.874 | 45.107 | 54.463 |
|  | 40.034 | 27.899 | 22.056 |
|  | 48.937 | 19.969 | 48.904 |
|  | 39.226 | 26.791 | 39.82 |
|  | 53.274 | 43.582 | 28.182 |

| Fig 6C The hyphal length of *C. albicans* grown on RPMI 1640 agar for 5 days | | |
| --- | --- | --- |
| Hyphal length (μm) | | |
| SN152 | ***cda2*Δ/Δ** | ***cda2*Δ/Δ+*CDA2*** |
| 2.036 | 1.311 | 3.23 |
| 2.406 | 1.414 | 2.786 |
| 2.221 | 1.568 | 2.947 |
| 2.545 | 1.288 | 2.726 |
| 2.54 | 1.455 | 3.057 |
| 2.599 | 2.198 | 2.945 |
| 2.962 | 1.066 | 2.575 |
| 2.874 | 1.597 | 2.683 |
| 2.863 | 1.818 | 2.32 |
| 2.321 | 1.985 | 3.063 |
| 2.506 | 1.929 | 2.694 |
| 2.679 | 1.481 | 2.617 |
| 2.6 | 1.171 | 2.915 |
| 2.487 | 1.655 | 2.552 |
| 2.622 | 1.593 | 2.647 |
| 2.278 | 1.677 | 2.505 |
| 2.727 | 1.047 | 2.141 |
| 2.37 | 1.968 | 2.035 |
| 2.278 | 1.969 | 2.15 |
| 2.72 | 1.592 | 2.209 |

| Fig 6E Transmission electron microscope image of the cell wall | | |
| --- | --- | --- |
| Cell-wall thickness (μm) | | |
| SN152 | ***cda2*Δ/Δ** | ***cda2*Δ/Δ+*CDA2*** |
| 0.068 | 0.163 | 0.122 |
| 0.068 | 0.211 | 0.122 |
| 0.082 | 0.177 | 0.109 |
| 0.095 | 0.136 | 0.109 |
| 0.108 | 0.193 | 0.11 |
| 0.095 | 0.17 | 0.073 |
| 0.108 | 0.164 | 0.109 |
| 0.135 | 0.165 | 0.123 |
| 0.081 | 0.219 | 0.115 |
| 0.127 | 0.136 | 0.095 |
| 0.116 | 0.173 | 0.108 |
| 0.081 | 0.211 | 0.103 |
| 0.058 | 0.122 | 0.096 |
| 0.095 | 0.154 | 0.095 |
| 0.108 | 0.136 | 0.079 |
| 0.122 | 0.201 | 0.13 |
| 0.108 | 0.252 | 0.121 |
| 0.068 | 0.149 | 0.112 |
| 0.095 | 0.174 | 0.15 |
| 0.082 | 0.154 | 0.073 |

| Fig 6G PI-positive RAW 264.7 cells co-incubated with *C. albicans* | | |
| --- | --- | --- |
| % of P^+^ cells | | |
| SN152 | ***cda2*Δ/Δ** | ***cda2*Δ/Δ+*CDA2*** |
| 36.1 | 7.8 | 52 |
| 34.9 | 8.5 | 30.2 |
| 34.3 | 5.3 | 24.4 |

| S1 Fig. A Time-growth curves of *C. albicans* SC5314 in YPD medium at 30 ℃ | | | |
| --- | --- | --- | --- |
| Time (h) | **OD_600_** | | |
|  | **Con** | **TA 16 μg/ml** | **TA 64 μg/ml** |
| 0 | 0.1 | 0.1 | 0.1 |
|  | 0.1 | 0.1 | 0.1 |
|  | 0.1 | 0.1 | 0.1 |
| 2 | 0.17 | 0.21 | 0.28 |
|  | 0.13 | 0.17 | 0.25 |
|  | 0.19 | 0.21 | 0.28 |
| 4 | 0.45 | 0.47 | 0.52 |
|  | 0.33 | 0.35 | 0.42 |
|  | 0.42 | 0.45 | 0.51 |
| 6 | 1.46 | 1.44 | 1.48 |
|  | 1.16 | 1.16 | 1.2 |
|  | 1.34 | 1.34 | 1.38 |
| 8 | 2.72 | 2.64 | 2.48 |
|  | 2.24 | 2.2 | 2.08 |
|  | 2.72 | 2.52 | 2.36 |
| 10 | 6.6 | 6.2 | 5.5 |
|  | 5.6 | 5.2 | 4.8 |
|  | 5.9 | 5.7 | 5.2 |
| 12 | 10.6 | 9.6 | 8.8 |
|  | 9.6 | 9 | 9 |
|  | 10.4 | 9.8 | 8 |
| 24 | 14 | 13.5 | 12.5 |
|  | 13 | 13.5 | 14.5 |
|  | 15 | 14 | 12.5 |

| S1 Fig. C The proliferation of yeast-blocked *efg1*Δ/Δ*cph1*Δ/Δ mutants in YPD medium 37 ℃ | | | |
| --- | --- | --- | --- |
| Time (h) | **OD_600_** | | |
|  | **Con** | **TA 16 μg/ml** | **TA 64 μg/ml** |
| 0 | 0.1 | 0.1 | 0.1 |
|  | 0.1 | 0.1 | 0.1 |
|  | 0.1 | 0.1 | 0.1 |
| 2 | 0.26 | 0.29 | 0.36 |
|  | 0.25 | 0.28 | 0.34 |
|  | 0.28 | 0.32 | 0.38 |
| 4 | 0.65 | 0.68 | 0.69 |
|  | 0.62 | 0.66 | 0.71 |
|  | 0.7 | 0.7 | 0.73 |
| 6 | 1.82 | 1.7 | 1.52 |
|  | 1.84 | 1.76 | 1.6 |
|  | 1.92 | 1.8 | 1.52 |
| 8 | 2.92 | 2.72 | 2.32 |
|  | 3.36 | 3.16 | 2.68 |
|  | 3.36 | 3.16 | 2.68 |
| 10 | 6.3 | 5.3 | 4 |
|  | 6.5 | 6.9 | 5.7 |
|  | 6.7 | 6.5 | 5.5 |
| 12 | 7.8 | 7.8 | 7.8 |
|  | 8.6 | 8.8 | 8 |
|  | 8.8 | 8.8 | 8 |
| 24 | 10.5 | 10 | 10 |
|  | 11.5 | 12 | 11 |
|  | 12 | 12.5 | 11 |

| S1 Fig. F *C. albicans* SC5314 were cultured in liquid RPMI 1640 and treated with double-distilled water or TA at 37 ℃ for 1 h,2 h and 3 h | | | | |
| --- | --- | --- | --- | --- |
| Time (h) | **Hyphal length (μm)** | | | |
|  | **Con** | **TA 0.5 μg/ml** | **TA 2 μg/ml** | **TA 8 μg/ml** |
| 1 | 2.824 | 12.329 | 8.248 | 4.302 |
|  | 10.455 | 3.875 | 4.386 | 1.361 |
|  | 6.833 | 1.532 | 13.647 | 8.605 |
|  | 7.565 | 4.795 | 6.315 | 10.906 |
|  | 6.202 | 1.937 | 1.532 | 6.734 |
|  | 5.648 | 5.812 | 1.937 | 4.138 |
|  | 2.166 | 6.883 | 3.425 | 4.906 |
|  | 2.055 | 2.74 | 5.648 | 5.61 |
|  | 6.985 | 6.985 | 5.349 | 2.453 |
|  | 7.052 | 4.166 | 4.332 | 5.852 |
|  | 1.532 | 0 | 8.844 | 7.962 |
|  | 0 | 5.522 | 5.582 | 13.086 |
|  | 13.085 | 10.83 | 8.304 | 9.138 |
|  | 4.906 | 11.981 | 4.952 | 7.377 |
|  | 4.563 | 13.767 | 3.401 | 3.492 |
|  | 6.454 | 4.386 | 1.924 | 3.063 |
|  | 10.406 | 3.063 | 1.359 | 5.479 |
|  | 3.663 | 3.425 | 2.883 | 2.055 |
|  | 8.87 | 9.54 | 3.397 | 0 |
|  | 10.368 | 8.93 | 10.893 | 7.052 |
|  | 12.828 | 7.809 | 8.266 | 5.522 |
|  | 5.892 | 6.746 | 3.397 | 9.253 |
|  | 4.362 | 2.74 | 3.039 | 8.658 |
|  | 8.641 | 0.969 | 1.519 | 6.7 |
|  | 6.358 | 2.824 | 6.828 | 3.663 |
|  | 4.021 | 4.905 | 5.307 | 1.924 |
|  | 2.759 | 2.151 | 3.962 | 3.663 |
|  | 10.904 | 8.276 | 7.924 | 2.721 |
|  | 9.655 | 7.756 | 0 | 5.61 |
|  | 12.044 | 4.302 | 11.565 | 3.469 |
|  | 2.069 | 3.401 | 3.042 | 9.451 |
|  | 2.487 | 7.004 | 5.852 | 5.181 |
|  | 6.849 | 3.967 | 7.606 | 4.563 |
|  | 2.824 | 9.668 | 5.181 | 1.924 |
|  | 3.425 | 9.252 | 11.9 | 4.302 |
|  | 10.83 | 4.563 | 6.7 | 12.954 |
|  | 2.47 | 8.869 | 12.907 | 6.937 |
|  | 0.685 | 2.721 | 8.304 | 6.454 |
|  | 9.189 | 2.041 | 6.122 | 2.805 |
|  | 7.281 | 4.302 | 4.302 | 8.87 |
| 2 | 23.177 | 19.884 | 6.126 | 14.558 |
|  | 16.917 | 24.362 | 27.406 | 17.21 |
|  | 22.467 | 18.553 | 12.499 | 11.644 |
|  | 28.381 | 9.838 | 17.861 | 13.417 |
|  | 21.1 | 7.309 | 5.648 | 29.87 |
|  | 15.634 | 10.999 | 21.011 | 28.878 |
|  | 29.42 | 22.092 | 23.378 | 22.705 |
|  | 20.707 | 10.683 | 13.457 | 20.823 |
|  | 15.022 | 18.59 | 25.352 | 25.716 |
|  | 18.944 | 18.38 | 16.695 | 19.74 |
|  | 26.983 | 31.034 | 12.064 | 6.803 |
|  | 15.768 | 24.904 | 16.552 | 16.58 |
|  | 20.876 | 23.947 | 11.374 | 11.044 |
|  | 16.121 | 15.453 | 11.744 | 15.161 |
|  | 6.746 | 23.024 | 32 | 17.246 |
|  | 7.565 | 7.658 | 6.543 | 14.383 |
|  | 8.017 | 16.237 | 8.043 | 11.704 |
|  | 10.83 | 7.409 | 17.131 | 12.347 |
|  | 3.492 | 10.959 | 15.757 | 11.15 |
|  | 6.78 | 13.869 | 16.165 | 14.754 |
|  | 17.341 | 14.558 | 9.515 | 11.074 |
|  | 6.498 | 28.048 | 9.54 | 17.007 |
|  | 15.768 | 27.991 | 11.973 | 14.717 |
|  | 27.139 | 19.361 | 29.172 | 20.812 |
|  | 18.199 | 8.87 | 25.892 | 8.192 |
|  | 10.113 | 17.344 | 12.63 | 24.762 |
|  | 4.11 | 14.935 | 27.474 | 18.418 |
|  | 19.946 | 7.933 | 20.465 | 16.327 |
|  | 20.364 | 16.327 | 14.024 | 23.408 |
|  | 19.105 | 13.4 | 8.87 | 7.102 |
|  | 15.316 | 21.266 | 14.92 | 9.475 |
|  | 10.633 | 15.619 | 15.513 | 17.317 |
|  | 34.439 | 11.15 | 14.35 | 15.939 |
|  | 22.891 | 23.924 | 12.264 | 19.775 |
|  | 4.166 | 18.695 | 13.503 | 16.355 |
|  | 12.63 | 14.562 | 5.181 | 12.488 |
|  | 14.449 | 16.048 | 17.344 | 20.088 |
|  | 8.219 | 6.849 | 15.287 | 9.253 |
|  | 12.348 | 13.492 | 19.03 | 15.527 |
|  | 30.02 | 6.498 | 13.698 | 21.906 |
| 3 | 39.736 | 48.533 | 30.516 | 26.721 |
|  | 34.687 | 37.434 | 35.938 | 47.136 |
|  | 12.835 | 17.061 | 19.19 | 22.436 |
|  | 20.69 | 38.15 | 47.355 | 48.635 |
|  | 21.779 | 27.092 | 46.688 | 25.674 |
|  | 26.808 | 24.734 | 39.928 | 43.746 |
|  | 27.245 | 46.108 | 28.098 | 43.54 |
|  | 49.702 | 36.873 | 18.568 | 27.948 |
|  | 35.648 | 23.427 | 26.047 | 18.379 |
|  | 39.256 | 27.228 | 20.226 | 19.946 |
|  | 27.61 | 42.115 | 25.499 | 27.704 |
|  | 21.277 | 38.095 | 36.677 | 47.355 |
|  | 25.203 | 29.886 | 20.734 | 39.191 |
|  | 42.719 | 26.801 | 23.732 | 32.777 |
|  | 26.677 | 16.127 | 12.544 | 40.831 |
|  | 22.768 | 25.472 | 25.925 | 20.051 |
|  | 49.034 | 39.775 | 32.148 | 27.397 |
|  | 39.903 | 48.189 | 45.339 | 12.63 |
|  | 30.168 | 49.168 | 28.409 | 41.056 |
|  | 25.352 | 22.613 | 15.867 | 18.114 |
|  | 34.754 | 38.844 | 34.687 | 27.423 |
|  | 33.5 | 47.86 | 48.775 | 26.461 |
|  | 25.399 | 16.495 | 27.414 | 29.963 |
|  | 41.697 | 10.576 | 29.737 | 23.732 |
|  | 30.055 | 17.529 | 23.648 | 34.128 |
|  | 47.253 | 25.862 | 48.24 | 26.17 |
|  | 42.527 | 20.859 | 50.051 | 27.899 |
|  | 26.744 | 40.972 | 26.171 | 26.073 |
|  | 27.991 | 30.949 | 18.944 | 24.051 |
|  | 13.4 | 12.404 | 31.507 | 31.938 |
|  | 42.055 | 18.396 | 40.295 | 23.81 |
|  | 13.699 | 19.134 | 30.226 | 25.562 |
|  | 19.91 | 32.649 | 27.624 | 29.775 |
|  | 26.598 | 41.05 | 17.223 | 33.5 |
|  | 50.671 | 37.377 | 25.399 | 25.904 |
|  | 35.597 | 42.582 | 37.856 | 15.287 |
|  | 45.513 | 31.98 | 28.319 | 20.465 |
|  | 27.092 | 27.277 | 20.419 | 21.959 |
|  | 31.3 | 16.034 | 25.562 | 35.269 |
|  | 47.096 | 36.885 | 24.848 | 18.317 |

| S3 Fig. C Inhibitory percentage of TA against *C. albicans* SN152, *cda2*Δ/Δ and *cda2*Δ/Δ+*CDA*2 | | |
| --- | --- | --- |
| *C. albicans* | **Inhibitory percentage (%)** | |
|  | **TA 0.5 μg/ml** | **TA 2 μg/ml** |
| SN152 | 50 | 49 |
|  | 58 | 66 |
|  | 40 | 69 |
| *cda2*Δ/Δ | 9 | 26 |
|  | 14 | 22 |
|  | 26 | 21 |
| *cda2*Δ/Δ+*CDA2* | 50 | 41 |
|  | 37 | 51 |
|  | 45 | 60 |
